# Sex differences in the association of prediabetes and type 2 diabetes with microvascular complications and function: The Maastricht Study

**DOI:** 10.1186/s12933-021-01290-x

**Published:** 2021-05-07

**Authors:** Rianneke de Ritter, Simone J. S. Sep, Carla J. H. van der Kallen, Marleen M. J. van Greevenbroek, Marit de Jong, Rimke C. Vos, Michiel L. Bots, Jos P. H. Reulen, Alfons J. H. M. Houben, Carroll A. B. Webers, Tos T. J. M. Berendschot, Pieter C. Dagnelie, Simone J. P. M. Eussen, Miranda T. Schram, Annemarie Koster, Sanne A. E. Peters, Coen D. A. Stehouwer

**Affiliations:** 1 Department of Internal Medicine, Maastricht University Medical Centre+, Maastricht, The Netherlands; 2 CARIM Cardiovascular Research Institute Maastricht, Maastricht University, Maastricht, The Netherlands; 3 Adelante, Centre of Expertise in Rehabilitation and Audiology, Hoensbroek, The Netherlands; 4 Julius Center for Health Sciences and Primary Care, University Medical Center Utrecht, Utrecht University, Utrecht, The Netherlands; 5 Leiden University Medical Center, Dept Public Health and Primary Care/LUMC-Campus, The Hague, The Netherlands; 6 Department of Clinical Neurophysiology, Maastricht University Medical Center+, Maastricht, The Netherlands; 7 Department of Ophthalmology, Maastricht University Medical Center+, Maastricht, The Netherlands; 8 Department of Epidemiology, Maastricht University, Maastricht, The Netherlands; 9 Heart and Vascular Centre, Maastricht University Medical Centre+, Maastricht, The Netherlands; 10 Department of Social Medicine, Maastricht University, Maastricht, The Netherlands; 11 CAPHRI Care and Public Health Research Institute, Maastricht University, Maastricht, The Netherlands; 12 The George Institute for Global Health, University of Oxford, Oxford, UK; 13 The George Institute for Global Health, University of New South Wales, Sydney, Australia

**Keywords:** Epidemiology, Type 2 diabetes, Sex, Sex difference, Women, Microvascular complications, Nephropathy, Neuropathy, Retinopathy

## Abstract

**Background:**

Women with type 2 diabetes are disproportionally affected by macrovascular complications; we here investigated whether this is also the case for microvascular complications and retinal microvascular measures.

**Methods:**

In a population-based cohort study of individuals aged 40–75 years (n = 3410; 49% women, 29% type 2 diabetes (oversampled by design)), we estimated sex-specific associations, and differences therein, of (pre)diabetes (reference: normal glucose metabolism), and of continuous measures of glycemia with microvascular complications and retinal measures (nephropathy, sensory neuropathy, and retinal arteriolar and venular diameters and dilatation). Sex differences were analyzed using regression models with interaction terms (i.e. sex-by- (pre)diabetes and sex-by-glycemia) and were adjusted for potential confounders.

**Results:**

Men with type 2 diabetes (but not those with prediabetes) compared to men with normal glucose metabolism, (and men with higher levels of glycemia), had significantly higher prevalences of nephropathy (odds ratio: 1.58 95% CI (1.01;2.46)) and sensory neuropathy (odds ratio: 2.46 (1.67;3.63)), larger retinal arteriolar diameters (difference: 4.29 µm (1.22;7.36)) and less retinal arteriolar dilatation (difference: − 0.74% (− 1.22; − 0.25)). In women, these associations were numerically in the same direction, but generally not statistically significant (odds ratios: 1.71 (0.90;3.25) and 1.22 (0.75;1.98); differences: 0.29 µm (− 3.50;4.07) and: − 0.52% (− 1.11;0.08), respectively). Interaction analyses revealed no consistent pattern of sex differences in the associations of either prediabetes or type 2 diabetes or glycemia with microvascular complications or retinal measures. The prevalence of advanced-stage complications was too low for evaluation.

**Conclusions:**

Our findings show that women with type 2 diabetes are not disproportionately affected by early microvascular complications.

**Supplementary Information:**

The online version contains supplementary material available at 10.1186/s12933-021-01290-x.

## Background

Type 2 diabetes is associated with an increased risk of both macro- and microvascular diseases [1]. Several studies have shown that type 2 diabetes is a stronger risk factor for macrovascular complications in women than in men [2]. In contrast, less is known about potential sex differences in the effects of diabetes on microvascular complications.

Studies that reported on sex differences in diabetes-associated microvascular complications are scarce and have shown inconsistent results [3–5]. An excess increased risk of microvascular complications associated with diabetes in women, compared with men, has been reported for vascular dementia [3]. No sex differences have been observed for chronic kidney disease [4], but in the same meta-analysis the excess risk for end stage renal disease associated with diabetes was higher in women than in men [4]. Finally, a prospective cohort study showed that only men with newly diagnosed diabetes and prediabetes had an increased risk of chronic kidney disease [5]. Most previous studies of diabetes-associated microvascular complications did not primarily focus on sex differences and were mostly restricted to one specific aspect of microvascular disease or to populations with type 2 diabetes only.

In view of these considerations, the aim of this study was to investigate sex differences in the associations of type 2 diabetes with classic microvascular complications, i.e. nephropathy, neuropathy and retinopathy. Additionally, to provide insights into the course of emergence of these potential sex disparities, we assessed sex differences in the associations of (pre)diabetes and measures of glycemia not only with microvascular complications but also with retinal microvascular diameters and function.

## Methods

### Study design and population

Data were used from The Maastricht Study, an observational prospective population-based cohort study. The rationale and methodology have been described previously [6]. In brief, The Maastricht Study focuses on the etiology, pathophysiology, complications, and comorbidities of type 2 diabetes and is characterized by an extensive phenotyping approach. Individuals aged between 40 and 75 years old at study baseline, and living in the southern part of the Netherlands, were eligible to participate. Participants were recruited through mass media campaigns, and from the municipal registries and the regional Diabetes Patient Registry via mailings. Recruitment was stratified according to known type 2 diabetes status, with an oversampling of individuals with type 2 diabetes, for reasons of efficiency. The present report includes cross-sectional data from the first 3451 participants, who completed the baseline survey between November 2010 and September 2013. Participants with other types of diabetes than type 2 diabetes or with a history of pancreatectomy were excluded (n = 41). The examinations of each participant were performed within a time window of three months. The study has been approved by the institutional medical ethical committee (NL31329.068.10) and the Minister of Health, Welfare and Sports of the Netherlands (Permit 131088–105234-PG). All participants gave written informed consent.

### Assessment of glucose metabolism status and measures of glycemia

To determine glucose metabolism status, all participants underwent a standardized 2-h (2-h) 75 g oral glucose tolerance test after fasting overnight. For safety reasons, participants using insulin or with a fasting glucose level above 11.0 mmol/L, as determined by a finger prick, did not undergo the oral glucose tolerance. For these individuals (n = 64), fasting glucose level and information about diabetes medication were used to determine glucose metabolism status. Glucose metabolism status was defined according to the WHO 2006 criteria into normal glucose metabolism (NGM), impaired fasting glucose, impaired glucose tolerance (combined as prediabetes), and type 2 diabetes [6]. Participants on blood glucose lowering medication were classified as having type 2 diabetes. Laboratory assessments of HbA1c, fasting glucose and of 2-h postload glucose were described elsewhere [6].

### Assessment of outcomes

*Nephropathy* was defined as an estimated glomerular filtration rate (eGFR) below 60 ml/min/1.73 m^2^, albuminuria, or both; and (or) a self-reported medical history of kidney transplantation or dialysis [6]. eGFR was calculated with the Chronic Kidney Disease Epidemiology Collaboration (CKD-EPI) equation based on both serum creatinine and serum cystatin C, measured in venous blood samples in the fasting state as described previously [6]. Presence of albuminuria was defined as an average urinary albumin excretion > 30 mg per 24 h measured in two 24-h urine samples as described previously [6].

*Sensory neuropathy* was defined as having neuropathic pain, impaired uni- or bilateral vibration perception, and (or) use of medication prescribed for neuropathic pain (gabapentine, pregabaline, duloxetine, amitriptyline, nortriptyline or carbamazepine, the latter only in individuals without a diagnosis of epilepsy) [6] (Appendix A). To determine the presence of neuropathic pain, a Dutch version of the DN-4 interview was used [6]. Vibration perception was measured three times with a Horwell Neurothesiometer (NTM) at the distal phalanx of the hallux of the right and left foot (Scientific Laboratory Supplies, Nottingham, UK) [6].

*Retinal measures* The presence of retinopathy was determined by use of fundus photography of both eyes. All fundus photographs are performed with a non-mydriatic auto fundus camera (Model AFC-230, Nidek, Gamagori, Japan) [6]. These numbers were too low to investigate sex-by- (pre)diabetes interactions, and therefore they are only presented in Table 1.

Additionally we determined retinal arteriolar and venular diameters from fundus photographs [7], presented as central retinal arteriolar equivalent and central retinal venular equivalent. Central retinal arteriolar equivalent and central retinal venular equivalent represent the equivalent single-vessel parent diameters for the 6 largest arterioles and largest venules in the region of interest, respectively. The calculations are based on the improved Knudtson-Hubbard formula [8]. More detailed information is described elsewhere [7]. The arteriolar and venular dilatation response to flicker light (an estimate of neurovascular coupling) is measured by use of the Dynamic Vessel Analyzer (Imedos Systems GmbH, Jena, Germany), as described in more detail elsewhere [7]. In short, per participant, we randomly measure the left or right eye. The participant is instructed to focus on the tip of a fixed needle inside the retinal camera while the fundus of the eye is examined under green measuring light. Straight arteriolar and venular segments of approximately 1.5 mm in length located 0.5–2.0 disc diameters from the margin of the optic disc in the temporal section are examined. After the specific vessel profile is recognized, its diameter is automatically and continuously measured for 150 s. A baseline recording of 50 s is followed by a 40-s flicker light exposure period and a subsequent 60-s recovery period [7]. The integrated Dynamic Vessel Analyzer software automatically calculates baseline diameter and percentage dilation. Baseline diameter is calculated as the average diameter size of the 20- to 50-s recording and is expressed in measurement units, where 1 measurement unit is equal to 1 μm of the Gullstrand eye [9]. Percentage dilation over baseline is based on the average dilation achieved at the time points 10 s and 40 s during the flicker stimulation period. Two regression lines are drawn (at intervals of 0– < 10 s and 10–40 s during flicker stimulation), and results are averaged to assess average percentage of dilation. The purpose of taking the average dilation is to account for interindividual variation in the curve shape during dilation.

### Statistical analyses

Statistical analyses were performed using SPSS version 25.0 for Windows (IBM SPSS, IBM Corp, Armonk, NY, USA). Population characteristics were described as mean ± standard deviation and median [interquartile range], for normally and non-normally distributed variables respectively or n (%) for discrete variables. Variables were log-transformed if residuals were skewed.

**Fig. 1:**
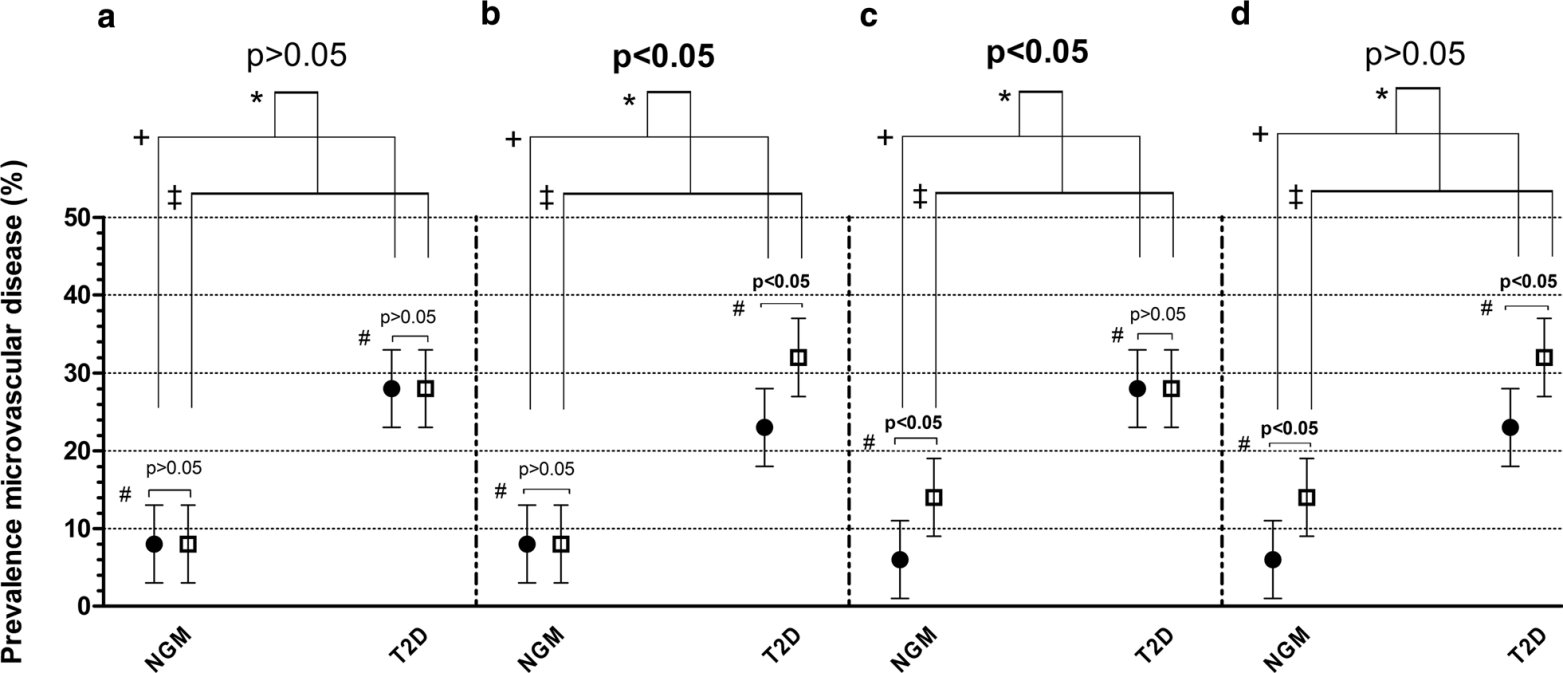
Microvascular disease: sex as a determinant versus sex-by-T2D interaction as a determinant. Simulation data to illustrate differences (**a**–**d**–described below) between sex as a determinant and sex-by-T2D interaction as a determinant for microvascular disease. Precise prevalences will vary in real data. Black circles represent women and white squares represent men. NGM: normal glucose metabolism; T2D: type 2 diabetes Microvascular disease: ^#^investigation of sex as a determinant; + women with T2D vs. women with NGM; ^‡^men with T2D vs. men with NGM; * investigation of sex-by-T2D interaction as a determinant. Statistically significantly differences (^#^ and/or * p < 0.05) are typed in bold. A: Sex is a determinant in neither NGM nor T2D; the sex-by-T2D interaction is not a determinant. B: Sex is a determinant in T2D but not in NGM; hence the sex-by-T2D interaction is a determinant. C: Sex is a determinant in NGM but not in T2D; hence (!) the sex-by-T2D interaction is a determinant. D: Sex is a determinant in both NGM and T2D; the sex-by-T2D interaction is not a determinant

Sex and the interaction of sex-by-(pre)diabetes need to be distinguished as potential determinants (Fig. 1). To investigate sex as determinant we used generalized linear models to estimate adjusted (model 2 as described below) sex-specific prevalences of nephropathy and sensory neuropathy, and adjusted means for retinal measures. Our main goal was to investigate sex-by- (pre)diabetes interaction as determinant. Therefore we used linear and logistic regression analyses to estimate sex-specific associations, and differences therein (i.e., interaction), of (1) prediabetes and type 2 diabetes (reference category: NGM) and of (2) continuous measures of glycemia (HbA1c, fasting glucose, 2-h postload glucose) with microvascular complications and retinal measures. To test for sex differences, interaction terms of sex by (pre)diabetes status and of sex by continuous measures of glycemia were incorporated into the models. Several sets of adjustments were used. Model 1 was adjusted for age. Model 2, the main model, was additionally adjusted for cardiovascular risk factors that have previously been associated with altered vessel- and inflammatory responses, and may therefore be potential confounders, i.e. waist circumference, triglyceride levels, total-to-HDL cholesterol ratio, systolic blood pressure, smoking status and use of antihypertensive and/or lipid-modifying medication. Model 3 was additionally adjusted for history of CVD, physical activity level, healthy diet score and educational level as additional potential confounders (definitions in Appendix A). For neuropathy-related variables, alcohol use was added to model 2, but not to model 3, as the healthy diet score also included alcohol use, and additional adjustments for height were performed either added in model 2 (mean neurothesiometer outcome) or taken into account within previous calculations (neuropathy–appendix A). For each potential confounder included, an interaction term (sex × potential confounder) was also incorporated in the same model, otherwise the adjustments made in the interaction models will not vary by sex as they do in the sex-specific models. For the interactions of sex with (pre)diabetes and of sex with measures of glycemia a p-interaction < 0.10 was considered statistically significant, as commonly used for statistical interaction testing [10]. Since the main goal of this study was to test for sex differences, both P-values < 0.05 and < 0.10 are shown and results are presented with a 95% confidence interval. For each dependent variable, a separate complete case analysis was performed. Sex-specific results are expressed as linear regression coefficients (betas, or geometric mean ratios in case of log-transformed variables) or logistic regression coefficients (odds ratios) (95%-CI) of the dependent variables. Sex differences are expressed as linear regression coefficients (women minus men mean difference, or women to men ratio of geometric mean ratios in case of log-transformed variable) or logistic regression coefficients (women to men ratio of odds ratios) (95%-CI) of the interaction terms.

To investigate the robustness of the results obtained by the above analyses, we did several sensitivity analyses. First, in all analyses, we used BMI instead of waist circumference. Second, we repeated all analyses after exclusion of premenopausal women (N = 338) and women in whom menopausal status was unclear (N = 55); analysis population N = 3,017). Third, we repeated analyses on neuropathy after exclusion of participants who used amitriptyline, nortriptyline, or carbamazepine (N = 15) as these medications can be prescribed for other indications than neuropathic pain. Finally, we additionally adjusted for metformin use in the analyses on neuropathy. Metformin use is related to vitamin B12 deficiency, which may cause neuropathy [11].

## Results

### Characteristics of the study population

The study population consisted of 1654 women (age 58.7 ± 8.2 years) and 1756 men (age 60.9 ± 8.1 years). Of these individuals, 1924 (57% women) had NGM, 511 (46% women) had prediabetes and 975 (32% women) had type 2 diabetes (Table 1).

**Table 1:** Study population characteristics according to sex and glucose metabolism status

	Women			Men		
	NGM n = 1103	Prediabetes n = 236	Type 2 diabetes n = 315	NGM n = 821	Prediabetes n = 275	Type 2 diabetes n = 660
Demographics						
Age (years)	57.3 ± 8.0	60.9 ± 7.9	62.0 ± 8.1	58.7 ± 8.4	62.1 ± 7.2	63.0 ± 7.5
Educational level						
Low, N (%)	338 (31.2)	91 (39.9)	181 (59.0)	168 (20.7)	84 (31.1)	258 (40.5)
Middle, N (%)	295 (27.2)	69 (30.3)	77 (25.1)	236 (29.1)	79 (29.3)	185 (29.0)
High, N (%)	451 (41.6)	68 (29.8)	49 (16.0)	408 (50.2)	107 (39.6)	194 (30.5)
Postmenopausal women, N (%)	810 (75.6)	186 (83.0)	265 (87.5)	NA	NA	NA
Hormone replacement therapy, N (%)	27 (2.5)	3 (1.3)	5 (1.6)	NA	NA	NA
Clinical characteristics						
Fasting glucose (mmol/l)	5.1 [4.8–5.4]	5.7 [5.3–6.2]	7.3 [6.4–8.1]	5.3 [5.0–5.6]	6.1 [5.7–6.4]	7.6 [6.9–8.8]
2-h postload glucose (mmol/l)^a^	5.4 [4.6–6.2]	8.7 [7.9–9.5]	14.4 [11.8–17.5]	5.4 [4.5–6.2]	8.1 [6.4–9.2]	14.4 [11.9–16.8]
HbA1c (%)	5.5 ± 0.3	5.7 ± 0.4	6.8 ± 1.0	5.4 ± 0.3	5.7 ± 0.4	7.0 ± 1.1
HbA1c (mmol/mol)	36.1 ± 3.7	38.8 ± 4.2	51.0 ± 11.2	36.0 ± 3.7	38.8 ± 4.7	52.7 ± 11.8
Fasting insulin (pmol/l)	50.1 [36.4–68.6]	71.9 [46.9–106.3]	87.5 [55.8–141.6]	57.8 [41.7–81.0]	72.5 [53.3–113.8]	83.9 [49.4–130.0]
Glucose-lowering medication, N (%)	NA	NA	237 (75.2)	NA	NA	529 (80.2)
Diabetes duration (years)	NA	NA	4.0 [1.0;9.0]	NA	NA	5.0 [2.0;12.0]
Cardiovascular risk factors						
History of CVD, N (%)	130 (12.0)	29 (12.4)	67 (22.0)	94 (11.5)	42 (15.4)	199 (31.1)
BMI (kg/m^2^)	25.1 ± 3.8	27.5 ± 4.7	30.4 ± 5.7	26.2 ± 3.2	28.0 ± 3.7	29.6 ± 4.6
Waist circumference (cm)	86.1 ± 10.2	93.2 ± 12.5	101.7 ± 14.4	96.3 ± 9.5	102.3 ± 10.5	107.8 ± 12.6
Office SBP (mmHg)	126.6 ± 16.9	134.2 ± 16.6	139.1 ± 17.6	136.1 ± 16.2	140.1 ± 16.9	144.0 ± 18.1
Office DBP (mmHg)	73.0 ± 9.5	75.7 ± 9.1	75.4 ± 9.0	78.2 ± 9.6	80.0 ± 9.5	78.1 ± 9.8
Antihypertensive medication use, N (%)	209 (18.9)	100 (42.4)	224 (71.1)	220 (26.8)	134 (48.7)	482 (73.0)
Total/HDL cholesterol ratio	3.3 ± 1.0	3.7 ± 1.2	3.5 ± 1.1	4.0 ± 1.3	4.0 ± 1.2	3.8 ± 1.1
Triglycerides (mmol/l)	1.02 [0.77–1.36]	1.34 [1.02–1.82]	1.54 [1.11–2.12]	1.13 [0.85–1.57]	1.35 [1.01–1.84]	1.53 [1.13–2.14]
Lipid-modifying medication use, N (%)	145 (13.1)	67 (28.4)	229 (72.7)	181 (22.0)	110 (40.0)	499 (75.6)
Smoking						
Never, N (%)	434 (39.8)	81 (34.8)	115 (37.6)	310 (37.9)	68 (25.0)	154 (24.0)
Former, N (%)	523 (47.9)	121 (51.9)	142 (46.4)	396 (48.5)	171 (62.9)	385 (60.0)
Current, N (%)	134 (12.3)	31 (13.3)	49 (16.0)	111 (13.6)	33 (12.1)	103 (16.0)
Alcohol use						
None, N (%)	192 (17.6)	56 (23.9)	154 (50.2)	70 (8.6)	24 (8.8)	135 (21.0)
Low, N (%)	568 (52.1)	105 (44.9)	109 (35.5)	550 (67.3)	165 (60.7)	369 (57.4)
High, N (%)	330 (30.3)	73 (31.2)	44 (14.3)	197 (24.1)	83 (30.5)	139 (21.6)
Physical activity						
Total self-reported physical activity (hours/week)	16.3 ± 8.1	16.1 ± 7.4	14.0 ± 7.7	13.3 ± 8.0	12.2 ± 7.8	11.3 ± 7.6
Dutch Healthy Diet Index	88.43 ± 13.66	86.93 ± 13.99	84.71 ± 13.91	80.49 ± 14.45	78.22 ± 14.95	78.03 ± 14.04
Microvascular complications and retinal measures						
Presence of microvascular disease^b^, N (%)	91 (11.4)	27 (15.1)	84 (32.8)	99 (16.5)	56 (28.3)	272 (51.5)
Nephropathy ^c^	45 (4.1)	14 (5.9)	57 (18.2)	57 (6.9)	32 (11.6)	170 (25.8)
eGFR < 60 ml/min/1.73 m^2^	20 (1.8)	6 (2.6)	30 (9.6)	13 (1.6)	14 (5.1)	60 (9.2)
eGRF (ml/min/1.73 m^2^)	90.07 ± 13.27	87.17 ± 14.22	84.17 ± 17.57	90.38 ± 13.26	86.15 ± 14.10	84.96 ± 17.14
Albuminuria (> 30 mg urinary albumin excretion per 24 h)	27 (2.6)	10 (4.6)	35 (12.4)	51 (6.7)	22 (8.6)	129 (21.7)
Albumin excretion (mg/24 h)	5.84 [3.67–9.35]	5.86 [3.87–10.01]	7.56 [4.76–15.32]	6.18 [3.76–10.25]	7.20 [4.47–11.35]	11.19 [6.00–25.21]
History of kidney transplantation, N (%)	2 (0.2)	0 (0.0)	0 (0.0)	1 (0.2)	0 (0.0)	0 (0.0)
History of hemodialysis, N (%)	1 (0.3)	0 (0.0)	0 (0.0)	1 (0.3)	0 (0.0)	0 (0.0)
Sensory neuropathy ^d^	136 (12.4)	47 (20.2)	92 (29.4)	96 (11.7)	43 (15.6)	222 (34.3)
Neuropathic pain, N (%)	74 (6.9)	28 (12.3)	59 (20.3)	45 (5.5)	17 (6.3)	112 (18.2)
Impaired unilateral vibration perception, N (%)	33 (3.4)	10 (5.1)	22 (7.8)	35 (4.9)	17 (7.5)	65 (11.7)
Impaired bilateral vibration perception, N (%)	20 (2.1)	7 (3.5)	15 (5.3)	20 (2.8)	9 (3.9)	70 (12.6)
Mean neurothesiometer outcome on the right and left first toe (Volt)	8.75 (6.25;12.60)	9.85 (7.00;14.25)	10.45 (7.74;16.35)	11.48 (7.40;16.58)	12.20 (8.95;18.95)	15.40 (9.85;23.43)
Retinopathy	1 (0.1)	0 (0.0)	9 (3.1)	0 (0.0)	1 (0.4)	35 (5.7)
Retinal microvasculature vessel diameters						
Central retinal arteriolar equivalent (µm)	145.44 ± 19.72	144.63 ± 20.67	144.49 ± 19.95	139.05 ± 20.13	138.31 ± 19.36	140.44 ± 21.52
Central retinal venular equivalent (µm)	216.44 ± 29.71	219.77 ± 32.92	218.54 ± 31.29	210.07 ± 31.51	212.91 ± 29.80	214.36 ± 33.06
Flicker light-induced arteriolar and venular dilation						
Flicker light-induced arteriolar dilation (%)	3.32 ± 2.81	2.84 ± 2.68	2.51 ± 2.69	3.46 ± 2.84	3.14 ± 2.80	2.20 ± 2.60
Flicker light-induced venular dilation (%)	4.05 ± 2.17	4.09 ± 2.22	3.86 ± 2.45	3.82 ± 2.17	4.01 ± 2.23	3.49 ± 2.18

T2DM: type 2 diabetes; CVD: cardiovascular disease. Data are expressed as mean ± standard deviation, median [interquartile range], or n (%), as appropriate

^a^missing data in 25% of individuals with T2DM per protocol

^b^Microvascular disease was defined as having retinopathy in one or both eyes, an estimated glomerular filtration rate (eGFR) < 60 ml/min/1.73 m^2^, albuminuria, and/or an impaired vibration perception of one or both first toes

^c^Nephropathy was defined as an eGFR < 60 ml/min/1.73 m^2^, albuminuria, or both; and/or a self-reported medical history of kidney transplantation or dialysis

^d^Diabetic sensory neuropathy was defined as having neuropathic pain and/or, impaired uni- or bilateral vibration perception

### Nephropathy

**Fig. 2:**
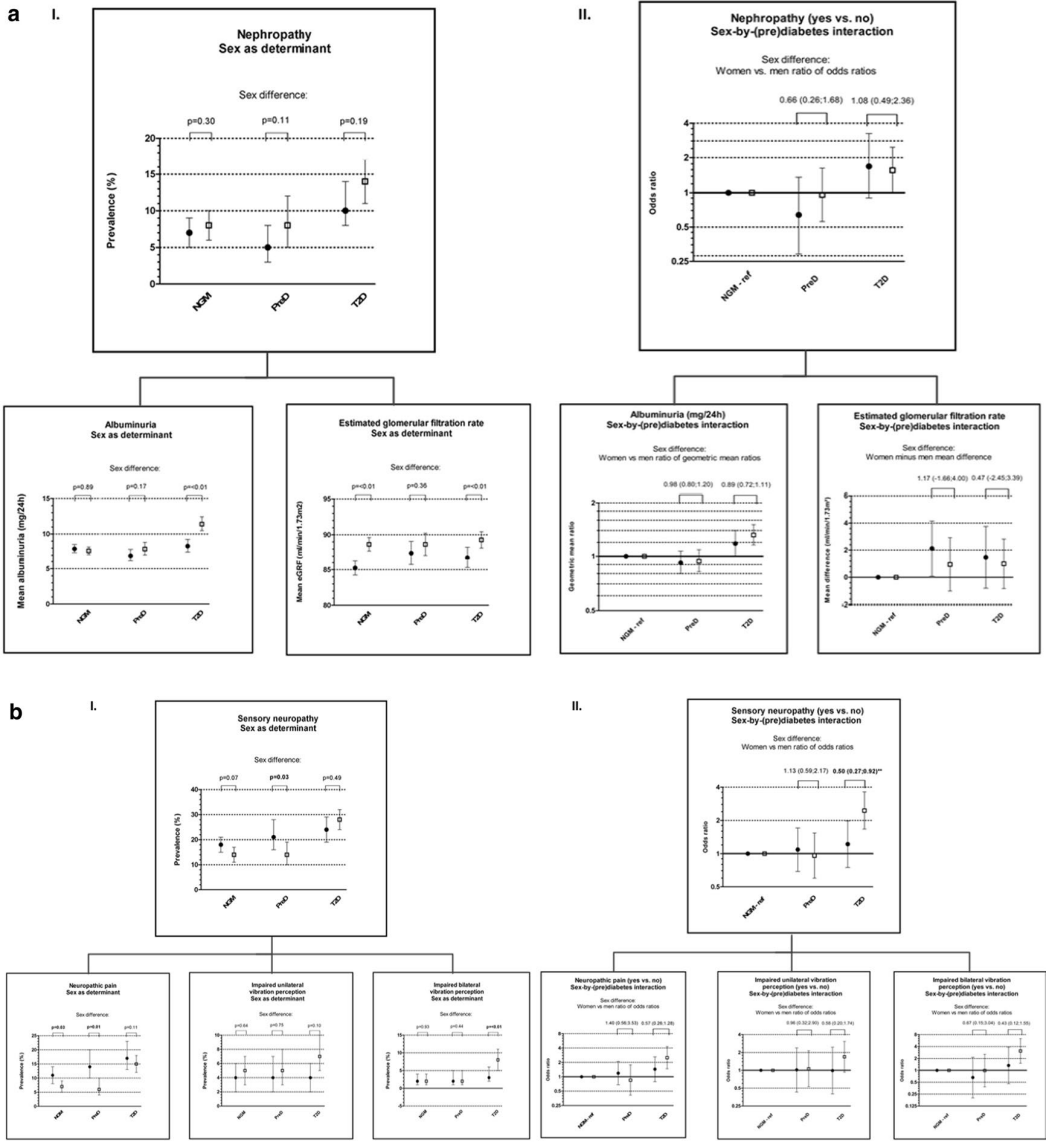

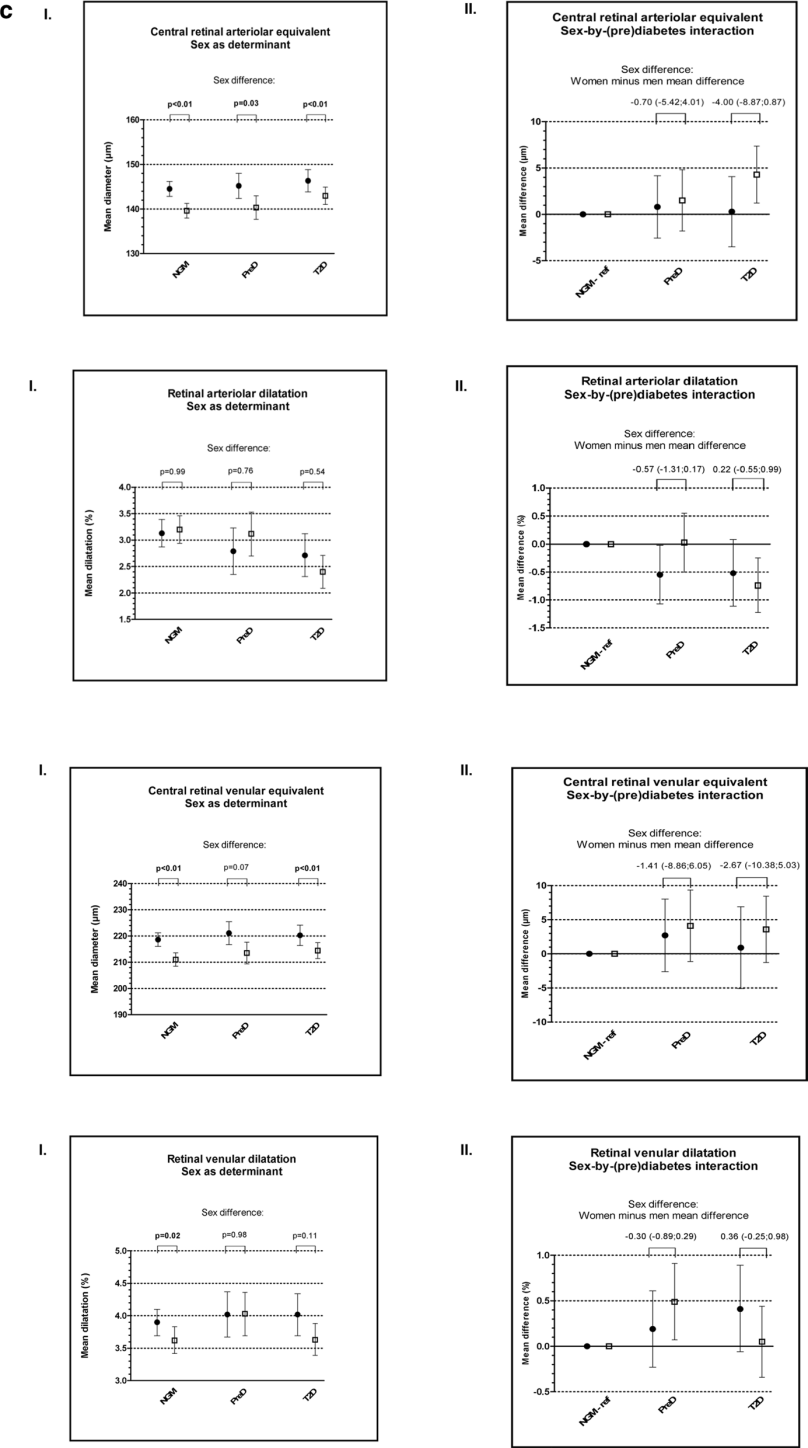
I.: Sex as determinant of nephropathy (**a**), sensory neuropathy (**b**) and retinal measures (**c**). The figure shows adjusted (model 2) sex-specific prevalences and corresponding 95%-CIs of nephropathy (**a**), sensory neuropathy (**b**), neuropathic pain (**b**) and impaired uni- and bilateral vibration perception (**b**) and means and corresponding 95%-CIs of albuminuria (**a**), estimated glomerular filtration rate (**a**) and the retinal diameter (**c**) and dilatation (**c**) in participants with a normal glucose metabolism, prediabetes or type 2 diabetes. Black circles represent women en white squares represent men. NGM: normal glucose metabolism, ref: reference group, preD: prediabetes, T2D: type 2 diabetes. Adjusted (model 2) differences between women and men (sex differences) are presented and statistically different sex differences (p < 0.05) are typed in bold. II.: Sex-by-(pre)diabetes as determinant of nephropathy (**a**), sensory neuropathy (**b**) and retinal measures (**c**). The figure shows adjusted (model 2) sex-specific odds ratios of nephropathy (**a**), sensory neuropathy (**b**), neuropathic pain (**b**) and impaired uni- and bilateral vibration perception (**b**), geometric mean ratios of albuminuria (**a**) and mean differences of estimated glomerular filtration rate (**a**) and the retinal diameter (**c**) and dilatation (**c**) between (pre)diabetes and normal glucose metabolism (reference category). NGM (ref) odds ratio is at 1.0 for women and men, but represents different prevalences for women and men (Fig. 1a–c-I). Black circles represent comparisons among women and white squares represent comparisons among men. PreD: prediabetes; T2D: type 2 diabetes. Results are expressed as adjusted linear or logistic regression coefficients and corresponding 95%-CIs. Differences between women and men (sex differences) are presented and statistically significantly different sex differences are typed in bold. ••p value < 0.05 • p value < 0.10

Sex as determinant: there were no statistically significant sex differences in the prevalences of nephropathy in participants with NGM, prediabetes and type 2 diabetes (Fig. 2a–I). Men, as compared to women, with type 2 diabetes (but not those with NGM or prediabetes) had a statistically significantly higher level of albuminuria (Fig. 2a–I). In contrast, women, as compared to men, with NGM and type 2 diabetes (but not those with prediabetes) had a statistically significantly lower eGFR (Fig. 2a–I).

Sex-by-(pre)diabetes interaction and sex-by-glycemia interaction as determinant: men with type 2 diabetes (but not those with prediabetes) compared to men with NGM, or men with higher levels of glycemia, had statistically significantly higher prevalences of nephropathy and higher levels of albuminuria. In women a similar pattern was seen, although this was statistically significant only for albuminuria, and generally not for nephropathy (Fig. 2a-I, II, Table 2, Additional file 1: Table S1, model 2). There were no statistically significant sex differences in the associations of prediabetes and type 2 diabetes or of continuous measures of glycemia with nephropathy, albuminuria or eGFR (Fig. 2a-II, Table 2, and Additional file 1: Table S1, model 2). Additional adjustments (model 3) did not materially change these results (Additional file 1: Table S1–2).

**Table 2:** Differences within and between sexes in mean differences in microvascular complications and retinal measures according to HbA1c, fasting glucose and 2-h postload glucose

	HbA1c (%) β, OR or GMR (95%-CI)		Fasting glucose (mmol/l) β, OR or GMR (95%-CI)		2-h postload glucose (mmol/l) β, OR or GMR (95%-CI)		Sex difference β, WM-OR or WM-GMR (95% CI)		
	Women	Men	Women	Men	Women	Men	HbA1c	Fasting glucose	2-h postload glucose
Nephropathy^*^ n = 2737 (Yes vs. no)	1.37 (1.05;1.79)	1.39 (1.18;1.64)	1.12 (0.96;1.32)	1.15 (1.06;1.25)	1.04 (0.98;1.11)	1.03 (0.99;1.08)	0.99 (0.72;1.35)	0.97 (0.82;1.16)	1.01 (0.94;1.09)
Albuminuria (mg/24 h)^†^ n = 2566	1.12 (1.03;1.22)	1.20 (1.14;1.27)	1.06 (1.01;1.11)	1.10 (1.07;1.13)	1.01 (0.99;1.02)	1.02 (1.01;1.03)	0.93 (0.84;1.03)	0.96 (0.91;1.02)	0.99 (0.97;1.01)
Estimated glomerular filtration rate (ml/min/1.73m^2^) n = 2713	− 0.10 (− 1.22;1.02)	0.14 (− 0.65;0.92)	0.66 (0.01;1.31)	0.39 (− 0.02;0.80)	0.17 (− 0.04;0.39)	0.13 (− 0.04;0.31)	− 0.24 (− 1.60;1.13)	0.27 (− 0.50;1.03)	0.04 (− 0.24;0.32)
Sensory neuropathy^*^ n = 2729 (Yes vs. no)	1.31 (1.04;1.63)	1.41 (1.21;1.64)	1.05 (0.93;1.19)	1.12 (1.03;1.21)	1.01 (0.96;1.06)	1.08 (1.04;1.13)	0.93 (0.71;1.22)	0.94 (0.81;1.09)	**0.93 •• (0.88;0.99)**
Neuropathic pain^*^ n = 2692 (Yes vs. no)	1.32 (1.03;1.69)	1.39 (1.16;1.66)	1.08 (0.94;1.23)	1.11 (1.01;1.22)	0.99 (0.93;1.05)	1.09 (1.04;1.15)	0.95 (0.70;1.29)	0.97 (0.82;1.14)	**0.90 •• (0.84;0.98)**
Impaired vibration perception^*^ n = 2384 Unilateral vs. no impaired vibration perception	1.04 (0.64;1.69)	1.16 (0.91;1.48)	0.95 (0.71;1.26)	1.06 (0.94;1.20)	1.05 (0.96;1.14)	1.04 (0.98;1.10)	0.89 (0.52;1.54)	0.89 (0.65;1.22)	1.01 (0.91;1.11)
Bilateral vs. no impaired vibration perception	1.84 (1.27;2.65)	1.42 (1.13;1.79)	1.32 (1.06;1.65)	1.12 (1.00;1.26)	1.05 (0.94;1.17)	1.10 (1.03;1.17)	1.29 (0.84;1.99)	1.18 (0.92;1.52)	0.95 (0.84;1.08)
Mean neurothesiometer outcome on the right and left first toe (Volt) ^†^ n = 2392	1.07 (1.03;1.12)	1.08 (1.05;1.12)	1.02 (0.99;1.04)	1.03 (1.02;1.05)	1.00 (1.00;1.01)	1.01 (1.00;1.02)	0.99 (0.94;1.04)	0.98 (0.96;1.01)	1.00 (0.99;1.01)
Central retinal arteriolar equivalent (µm) n = 2394	− 0.15 (− 1.94;1.64)	1.51 (0.20;2.82)	− 0.28 (− 1.32;0.75)	0.31 (− 0.36;0.99)	− 0.23 (− 0.59;0.13)	0.32 (0.02;0.63)	− 1.66 (− 3.88;0.56)	− 0.59 (− 1.83;0.64)	− **0.55 •• (**− **1.02;**− **0.08)**
Central retinal venular equivalent (µm) n = 2394	1.41 (− 1.42;4.24)	1.24 (− 0.83;3.31)	0.39 (− 1.24;2.03)	− 0.23 (− 1.30;0.84)	− 0.28 (− 0.85;0.29)	0.12 (− 0.36;0.60)	0.17 (− 3.34;3.68)	0.63 (− 1.33;2.58)	− 0.40 (− 1.14;0.35)
Retinal arteriolar dilatation (%) n = 1878	− 0.35 (− 0.63;− 0.07)	− 0.41 (− 0.62;− 0.20)	− 0.25 (− 0.40;− 0.09)	− 0.17 (− 0.28;− 0.07)	0.00 (− 0.06;0.06)	− 0.05 (− 0.10;0.00)	0.07 (− 0.28;0.42)	− 0.08 (− 0.26;0.11)	0.05 (− 0.02;0.13)
Retinal venular dilatation (%) n = 1908	− 0.02 (− 0.25;0.21)	− 0.11 (− 0.29;0.06)	0.00 (− 0.13;0.12)	− 0.06 (− 0.14;0.03)	0.03 (− 0.02;0.08)	0.00 (− 0.04;0.04)	0.09 (− 0.19;0.38)	0.05 (− 0.10;0.20)	0.03 (− 0.03;0.09)

Sex-specific differences (main model: model 2) are expressed as linear or logistic regression coefficients (95%-CI) of the dependent variables, which indicate mean differences (βs), odds ratios (ORs) or geometric mean ratios (GMRs) in microvascular complications and retinal measures per one percent point increase in HbA1c or per one mmol/l increase in fasting glucose or 2-h postload glucose.

*ORs. † GMRs

Differences between sexes (main model: model 2) are expressed as linear or logistic regression coefficients (95%-CI) of the interaction terms sex*HbA1c, sex*fasting glucose and sex*2-h postload glucose, which indicate differences between women and men in mean differences (βs), women to men ratio of odds ratios (WM-ORs) or women to men ratio of geometric mean ratios (WM-GMRs) in microvascular complications and retinal measures per one percent point increase in HbA1c or per one mmol/l increase in fasting glucose or 2-h postload glucose. *WM-ORs. †WM-GMRs. Statistically significant differences between the sexes are typed in bold. ••p value < 0.05 • p value < 0.10

### Sensory neuropathy

Sex as determinant: women, as compared to men, with prediabetes (but not those with NGM or type 2 diabetes) had a statistically significantly higher prevalence of sensory neuropathy (Fig. 2b-I). Women, as compared to men, with NGM and prediabetes (but not those with type 2 diabetes) had a statistically significantly higher prevalence of neuropathic pain (Fig. 2b-I). In contrast, men, as compared to women, with type 2 diabetes (but not those with NGM or prediabetes), had a statistically significantly higher prevalence of impaired bilateral vibration perception (Fig. 2b-I).

Sex-by-(pre)diabetes interaction and sex-by-glycemia interaction as determinant: men with type 2 diabetes (but not those with prediabetes) compared to men with NGM, or men with higher levels of glycemia, had statistically significantly higher prevalences of sensory neuropathy, neuropathic pain and impaired bilateral vibration perception, and a higher level of the mean neurothesiometer outcome. In women these associations were numerically in the same direction, but generally not statistically significant (Fig. 2b-I, II, Table 2, Additional file 1: Table S1, model 2). Type 2 diabetes (but not prediabetes) was less strongly associated with sensory neuropathy in women than in men (women vs. men ratio of odds ratios (model 2): 0.50 (0.27;0.92)) (Fig. 2b-II, Additional file 1: Table S1, model 2). In contrast, there were no statistically significant sex differences in the associations of prediabetes and type 2 diabetes with neuropathic pain, uni- or bilateral impaired vibration perception or the mean neurothesiometer outcome (Fig. 2b-II, Additional file 1: Table S1, model 2). Additionally, there were no statistically significant sex differences in the associations of HbA1c and fasting glucose with sensory neuropathy, neuropathic pain, impaired uni- or bilateral vibration perception or the mean neurothesiometer outcome. In contrast, 2-h postload glucose was less strongly associated with sensory neuropathy and neuropathic pain (but not with uni- or bilateral impaired vibration perception or the mean neurothesiometer outcome) in women than in men (women vs. men ratio of odds ratios (model 2): 0.93 (0.88; 0.99) and 0.90 (0.84;0.98), respectively) (Table 2). Additional adjustments (model 3) did not materially change these results (Additional file 1: Table S1–2).

### Retinal measures

Sex as determinant: men, as compared to women, and regardless of glucose metabolism status, had a statistically significantly smaller central retinal arteriolar equivalent, while arteriolar dilatation did not differ statistically significantly (Fig. 2c-I). Men, as compared to women, with NGM and type 2 diabetes (but not those with prediabetes) had a smaller central retinal venular equivalent. Venular dilatation was statistically lower in men, as compared to women, in NGM, but not in prediabetes or type 2 diabetes (Fig. 2c-I).

Sex-by-(pre)diabetes interaction and sex-by-glycemia interaction as determinant: men with type 2 diabetes (but not those with prediabetes) compared to men with NGM, or men with higher levels of glycemia, had a statistically significantly larger central retinal arteriolar equivalent and less retinal arteriolar dilatation. In women these associations were numerically in the same direction, but generally not statistically significant (Fig. 2c–I, II; Table 2, Additional file 1: Table S1, model 2). Associations with regard to venular diameters and dilatation were generally not statistically significant (Fig. 2c–I, II; Table 2, Additional file 1: Table S1, model 2). There was no consistent pattern of sex differences in the associations of prediabetes, type 2 diabetes and continuous measures of glycemia with central retinal arteriolar and venular equivalent or with retinal arteriolar and venular dilatation (Fig. 2c-II, Table 2 and Additional file 1: Table S1, model 2). Additional adjustments (model 3) did not materially change these results (Additional file 1: Table S1–2).

### Sensitivity analyses

Results were not materially changed by using BMI instead of waist circumference (Additional file 1: Table S3); by excluding premenopausal women (Additional file 1: Table S4); or (for neuropathy) by excluding individuals who reported using amitriptyline, nortriptyline or carbamazepine (data not shown). After additional adjustment for metformin use, the observed sex difference to the disadvantage of men in the association of type 2 diabetes with sensory neuropathy was attenuated and no longer statistically significant. Results with regard to neuropathic pain, uni- and bilateral vibratory sense and the mean neurothesiometer were not materially changed (Additional file 1: Table S5).

## Discussion

The main finding of this study is that there was no consistent pattern of sex differences in the associations of glucose metabolism status and glycemia with microvascular complications or retinal microvascular measures (i.e. no consistent sex-by- (pre)diabetes or sex-by-glycemia interaction). Therefore, our results indicate that women are not disproportionally affected by microvascular complications of diabetes, which is in contrast with sex differences in the effects of diabetes on macrovascular complications [2].

With respect to nephropathy, the absence of sex-by- (pre)diabetes and sex-by-glycemia interactions is in line with results from a meta-analysis that reported no significant sex difference in the relative risk of nephropathy associated with diabetes [4]. This same meta-analysis did report a higher relative risk of end stage renal disease associated with diabetes in women than in men, and it was suggested that female sex may accelerate disease progression [4]. We cannot exclude this possibility as our study population had no cases of end stage renal disease (an outcome that is rare at the population level). Additionally, we observed no sex-by- (pre)diabetes or sex-by-glycemia interactions with regard to albuminuria or eGFR. Previous studies focused solely on sex differences in populations with type 2 diabetes (i.e. sex as determinant) and showed inconsistent results [12–17]. In these diabetic populations, male sex has been observed to be a risk factor for the development of persistent micro- and macroalbuminuria [13, 14], which is in line with our findings. In addition, eGFR decline has been observed to be greater in men [15] than in women, but the reverse [16], and no sex differences [17], have also been observed. In our study, eGFR was lower in women than in men, regardless of glucose metabolism status. Taken together, our and previous findings suggest that men, compared to women, with type 2 diabetes are at higher risk of developing albuminuria; for lower eGFR there is no consistent pattern. For neither albuminuria nor eGFR is there clear evidence for a sex-by- (pre)diabetes interaction. It should be noted that in non-diabetic populations premenopausal women are protected from renal disease; this protection is lost after menopause and in the presence of diabetes [12]. As women in our study were generally postmenopausal, we cannot exclude a sex-by- (pre)diabetes interaction in premenopause.

With respect to neuropathy, we observed a sex-by-diabetes interaction to the disadvantage of men. Although sex was not a statistically significant determinant in either NGM or type 2 diabetes, the interaction was nonetheless statistically significant because of a contrast between the sexes in highest observed prevalences in type 2 diabetes vs. NGM (prevalence in type 2 diabetes was higher in men than in women, while the reverse was observed in NGM). However, this observed sex difference to the disadvantage of men was attenuated and no longer statistically significant after additional adjustment for metformin use (as a proxy for low vitamin B12 levels). Additionally, no consistent pattern of sex-by- (pre)diabetes and sex-by-glycemia interactions was observed in the associations with the other neuropathy-related variables, i.e. neuropathic pain, impaired uni- or bilateral vibration perception or the mean neurothesiometer outcome, nor was there a sex-by-glycemia interaction in the association with neuropathy. This is in line with previous results from the Maastricht Study, which showed that associations of (pre)diabetes with motor and sensory nerve function (assessed by electromyography) do not differ between the sexes [18]. Additionally, results from our group showed that associations between (pre)diabetes and measures of glycemia with 24-h electrocardiogram-derived lower heart rate variability, as marker for autonomic neuropathy, do not differ between the sexes either [19]. Therefore, we conclude that the current and previous [18, 19] findings, taken together, generally indicate that sex is not a determinant of, and that there is no sex-by- (pre)diabetes interaction with regard to, neuropathy.

Previous studies have looked not at the sex-by-diabetes interaction but did look at sex as a determinant of neuropathy among individuals with type 2 diabetes, and have not reported consistent results [20–30]. Some studies reported more severe neuropathy [20, 21, 23] and a higher prevalence of neuropathy in men than in women [20–25]. However, a higher prevalence in women than men has also been reported [26]; women also more often and more intensely experience neuropathic pain [23, 27]. Additionally, some studies reported no sex difference in the prevalence of neuropathy [28–30]. Although diabetes (type 1 and 2 together) has been shown to confer a nearly 2.5 times higher relative risk of lower extremity amputation in men than in women [31], this does however not necessarily indicate a sex-by-diabetes interaction with regard to neuropathy, since foot ulcer, infection and peripheral vascular disease, in addition to neuropathy, are also major risk factors for diabetes-associated lower extremity amputation [32]. In sum, our findings generally indicate that sex is not a determinant of neuropathy. We observed a sex-by-diabetes interaction, to the disadvantage of men, but the lack of a sex-by-glycemia interaction and the absence of sex-by- (pre)diabetes interactions with other measures of neuropathy [18, 19] suggests that this result could be a chance finding. It should however again be noted that our population was relatively healthy, and we cannot exclude such interactions with regard to progression of neuropathy.

Diabetic retinopathy is by definition [33] rare among individuals without diabetes, and the usefulness of analyses of sex-by- (pre)diabetes interactions is therefore moot. Most previous studies therefore focused on sex as a determinant in populations with type 2 diabetes, and findings again have not been consistent [34–39]. In a large pooled analysis, the prevalence of diabetic retinopathy was similar in men and women [38]. However, in other studies, a higher prevalence and severity, and faster progression, of retinopathy among men than among women [34–37] have been reported, but the opposite has also been observed [39]. With regard to retinal measures, which are thought to reflect very early changes which predispose to retinopathy, we again observed no consistent sex-by- (pre)diabetes or sex-by-glycemia interactions. In sum, the effect of diabetes on retinopathy appears to be similar in both sexes, but again we cannot exclude sex differences in later phases of retinopathy.

The above results contrast with findings on sex-by- (pre)diabetes interactions with macrovascular complications [2]. It has been hypothesized that women’s cardiovascular risk factors deteriorate to a greater extent in the transition from normoglycemia to type 2 diabetes, possibly due to sex differences in body composition and fat distribution [2]. Consequently, women's vasculature may experience a prolonged exposure to metabolic dysfunction prior to diagnosis of type 2 diabetes and this could party explain women’s excess risk of diabetes-associated macrovascular complications [2]. Presumably, these sex differences do not, or to a lesser extent, influence microvascular complications.

Strengths of our study include its population-based design combined with oversampling of individuals with type 2 diabetes, which enables an accurate comparison of individuals with and without type 2 diabetes. Additionally, this study benefits from a large sample size and the detailed phenotypic assessment. The study also has some limitations. First, the data were cross-sectional; therefore, we cannot exclude reverse causality between (pre)diabetes and microvascular complications; however, we do not expect this to affect the investigated sex differences. Second, we could not assess sex-by- (pre)diabetes interaction with regard to advanced stages of microvascular complications, as these were rare in this population.

## Conclusions

In conclusion, there was no consistent pattern of sex differences in the associations of glucose metabolism status and glycemia with early microvascular complications. Thus, these results are consistent with the concept that diabetes does not confer a greater risk of microvascular complications among women than men. However, we cannot exclude such interactions with regard to progression of microvascular complications.

### Supplementary Information

**Additional file 1: Table S1.** Differences within and between sexes in mean differences in microvascular complications and retinal measures according to glucose metabolism status.** Table S2.** Differences within and between sexes in mean differences in microvascular complications and retinal measures according to HbA1c, fasting glucose and 2-h postload glucose (all models).** Table S3.** Differences between sexes in mean differences in microvascular complications and retinal measures according to glucose metabolism status – with BMI used instead of waist circumference.** Table S4.** Differences within and between sexes in mean differences in microvascular complications and retinal measures according to glucose metabolism status – premenopausal women excluded.** Table S5.** Differences within and between sexes in mean differences in neuropathy according to glucose metabolism status – with additional adjustment for metformin use (model 2 and 3).

## Data Availability

The datasets used and/or analysed during the current study are available from the corresponding author on reasonable request.

## Appendix A: Assessment of neuropathy, covariates and population characteristics

### Neuropathy

The DN-4 interview to determine the presence of neuropathic pain consists of seven items grouped into two questions, which describe the neuropathic pain (burning, painful cold, electric shocks) and its associated abnormal sensations (tingling, pins and needles, numbness, itching). A score greater than 3 was considered indicative of neuropathic pain [6].

Vibration perception was measured three times with a Horwell Neurothesiometer (NTM) at the distal phalanx of the hallux of the right and left foot (Scientific Laboratory Supplies, Nottingham, UK) [6]. The mean of the three NTM scores was calculated for each foot. However, if the coefficient of variation of the three measurements exceeded 40%, the outlying NTM score was excluded and the mean of the remaining two values was computed.

Normal vibration perception thresholds were estimated in a healthy subpopulation of the Maastricht Study. These individuals all have NTM data available and did not have (poly)neuropathy or diseases which predispose to (poly)neuropathy. (Poly)neuropathy, as exclusion criteria, was defined as either self-reported neuropathic pain (DN4-interview score > 3), and/or a severely asymmetric vibration perception (i.e. an extreme difference (values < or > 2SD) between the right and the left NTM score). The following diseases which predispose to neuropathy were identified: prediabetes, type 2 diabetes, an ankle brachial index < 0.90 in either leg, alcoholism (defined as > 21 glasses per week for either men or women [40]), reduced kidney function (defined as either as a CKD-EPI below 45 ml/min/1.73m2, kidney transplantation or dialysis), or severe movement limitations (defined as having difficulty walking 500 m or walking up the stairs based on the SF-36 questionnaire).

In statistical analyses, the NTM variable was log-transformed due to a skewed distribution. Linear regression analyses were used to determine the 97.5th percentile for the log-transformed NTM score as a function of sex and height [41], the same cases were identified irrespective of the score of which leg was used as an outcome measure. Therefore, based on the estimations of the right leg, the 97.5 percentile according to sex and height was computed for each individual in the full dataset. An impaired vibration perception was categorized in unilateral or bilateral vibration perception, and defined as a log-transformed NTM score greater than the predicted value in one leg or either leg, respectively.

### Covariates and population characteristics

We determined waist circumference, BMI, triglyceride levels, total cholesterol, HDL cholesterol, systolic and diastolic office blood pressure and fasting plasma levels of insulin as described elsewhere [6]. We assessed glucose-lowering, lipid-modifying and antihypertensive medication use, as well as postmenopausal hormone replacement therapy, during a medication interview where generic name, dose, and frequency were registered [6]. We used a questionnaire to assess age (years), sex, prior CVD (defined as a history of any of the following conditions: myocardial infarction, cerebrovascular infarction or hemorrhage, percutaneous artery angioplasty of, or vascular surgery on, the coronary, abdominal, peripheral or carotid arteries), smoking status (never, current, former), alcohol use (none, low (women ≤ 7 glasses per week; men ≤ 14 glasses per week), high (women ≤ 7 glasses per week; men ≤ 14 glasses per week)), adherence to the Dutch dietary guidelines and the indication of diet quality (based on fourteen out of fifteen components of the Dutch Healthy Diet index 2015, as information on filtered coffee intake was not collected [42], calculated from a validated food frequency questionnaire [43]; the total score ranges between 0 (no adherence) and 140 (complete adherence), educational level (low, intermediate, high), physical activity level (hours of moderate to vigorous physical activity per week) and postmenopausal status in women [6]. Finally, fundus photography of both eyes was performed to determine the presence of diabetic retinopathy, as described previously [6].

## Abbreviations

NGM: Normal glucose metabolism
eGFR: Estimated glomerular filtration rate
NTM: Neurothesiometer
