# Supplementary material for: Sex differences in the association of prediabetes and type 2 diabetes with microvascular complications and function: The Maastricht Study
Source: Cardiovasc Diabetol. 2021 May 7;20:102. doi: 10.1186/s12933-021-01290-x (PMC8106227; doi:10.1186/s12933-021-01290-x)
Supplement: Supplementary file 1 — Additional file 1: Table S1. Differences within and between sexes in mean differences in microvascular complications and retinal measures according to glucose metabolism status. Table S2. Differences within and between sexes in mean differences in microvascular complications and retinal measures according to HbA1c, fasting glucose and 2-h postload glucose (all models). Table S3. Differences between sexes in mean differences in microvascular complications and retinal measures according to glucose metabolism status – with BMI used instead of waist circumference. Table S4. Differences within and between sexes in mean differences in microvascular complications and retinal measures according to glucose metabolism status – premenopausal women excluded. Table S5. Differences within and between sexes in mean differences in neuropathy according to glucose metabolism status – with additional adjustment for metformin use (model 2 and 3). [file 12933_2021_1290_MOESM1_ESM.docx]

**Additional file**

**Additional file 1: Table S1: Differences within and between sexes in mean differences in microvascular complications and retinal measures according to glucose metabolism status**

|  |  | Prediabetes  β, OR or GMR  (95%-CI) | | Type 2 diabetes  β, OR or GMR  (95%-CI) | | Sex difference,  β, WM-OR or WM-GMR (95% CI) | |
| --- | --- | --- | --- | --- | --- | --- | --- |
|  |  | Women | Men | Women | Men | Prediabetes | Type 2 diabetes |
| Nephropathy^*^ n=2746  (Yes vs. no)    Albuminuria (mg/24h)^†^  n=2573  Estimated glomerular  filtration rate (ml/min/1.73m^2^)  n=2722 | Model 1  Model 2  Model 3  Model 1  Model 2  Model 3  Model 1  Model 2  Model 3 | 1.10 (0.54;2.22)  0.64 (0.29;1.37)  0.64 (0.30;1.39)  1.03 (0.89;1.19) 0.92 (0.80;1.07) 0.93 (0.80;1.08)  0.51  (-1.48;2.50) 2.12 (0.09;4.15) 2.12 (0.10;4.15) | 1.46 (0.87;2.43)  0.96 (0.56;1.65)  0.99 (0.58;1.71)  1.11 (0.97;1.29) 0.94 (0.82;1.09) 0.96 (0.83;1.11)  -0.85  (-2.81;1.11) 0.95  (-1.02;2.92) 0.87  (-1.11;2.84) | 3.93 (2.41;6.40)  1.71 (0.90;3.25)  1.72 (0.90;3.31)  1.40 (1.22;1.61) 1.18 (1.00;1.40) 1.20 (1.02;1.42)  -1.74  (-3.60;0.12) 1.47  (-0.81;3.75) 1.58  (-0.71;3.87) | 3.52 (2.44;5.08)  1.58 (1.01;2.46)  1.58 (1.00;2.47)  1.82 (1.62;2.03) 1.32 (1.16;1.51) 1.34 (1.17;1.53)  -1.93  (-3.46;-0.40) 1.00  (-0.83;2.82) 1.12  (-0.72;2.95) | 0.75 (0.31;1,80)  0.66 (0.26;1.68)  0.65 (0.25;1.65)    0.92 (0.75;1.13) 0.98 (0.80;1.20) 0.97 (0.79;1.19)  1.36  (-1.43;4.15) 1.17  (-1.66;4.00)  1.26  (-1.57;4.09) | 1.12 (0.61;2.06)  1.08 (0.49;2.36)  1.09 (0.50;2.41)  **0.77 •• (0.65;0.92)** 0.89 (0.72;1.11)  0.90 (0.72;1.11)  0.19  (-2.22;2.60) 0.47  (-2.45;3.39) 0.47  (-2.46;3.40) |
|  |  |  |  |  |  |  |  |
| Sensory neuropathy^*^  n=2736 (Yes vs. no)    Neuropathic pain^*^ n=2699 (Yes vs. no)    Impaired vibration perception^*^ n=2390  Unilateral vs. no impaired vibration perception  Bilateral vs. no impaired vibration perception  Mean neurothesiometer outcome on the right and left first toe (Volt)^†^  n=2398 | Model 1  Model 2  Model 3  Model 1  Model 2  Model 3  Model 1  Model 2  Model 3  Model 1  Model 2  Model 3  Model 1  Model 2  Model 3 | 1.54 (1.02;2.33) 1.09 (0.69;1.71)  1.12 (0.71;1.75)  1.75 (1.04;2.94)  1.19 (0.68;2.10)  1.24 (0.70;2.19)  1.42 (0.64;3.16)  1.02 (0.43;2.40)  1.01 (0.43;2.38)  0.74 (0.24;2.27)  0.66 (0.20;2.15)  0.72 (0.22;2.33)  1.03 (0.95;1.12)  1.04 (0.96;1.12)  1.04 (0.97;1.12) | 1.13 (0.72;1.78)  0.96 (0.60;1.54)  0.90 (0.56;1.46)  1.05 (0.52;2.14)  0.85 (0.41;1.77)  0.84 (0.41;1.76)  1.24 (0.63;2.44)  1.06 (0.53;2.14)  1.03 (0.51;2.08)  1.22 (0.48;3.07)  0.99 (0.38;2.55)  0.84 (0.32;2.20)  1.04 (0.96;1.13)  1.03 (0.96;1.11)  1.03 (0.95;1.11) | 2.52 (1.77;3.60) 1.22 (0.75;1.98)  1.33 (0.82;2.15)  3.14 (2.03;4.85)  1.44 (0.79;2.62)  1.63 (0.89;2.97)  1.82 (0.92;3.60)  0.99 (0.40;2.46)  0.95 (0.39;2.34)  1.50 (0.68;3.31)  1.33 (0.46;3.85)  1.34 (0.46;3.92)  1.11 (1.03;1.19)  1.08 (0.99;1.17)  1.07 (0.99;1.17) | 3.34 (2.44;4.58) 2.46  (1.67;3.63)  2.37 (1.60;3.50)  3.92 (2.52;6.11)  2.51 (1.47;4.28)  2.42 (1.41;4.15)  2.09 (1.27;3.43)  1.69 (0.93;3.08)  1.61 (0.89;2.93)  4.31 (2.35;7.91)  3.10 (1.51;6.35)  2.98 (1.43;6.21)  1.19 (1.12;1.27)  1.13 (1.06;1.22)  1.13 (1.06;1.21) | 1.36 (0.74;2.52)  1.13 (0.59;2.17)  1.23 (0.64;2.38)  1.67 (0.69;4.01)  1.40 (0.56;3.53)  1.47 (0.58;3.72)  1.14 (0.40;3.26)  0.96 (0.32;2.90)  0.98 (0.32;2.98)  0.61 (0.14;2.59)  0.67 (0.15;3.04)  0.85 (0.19;3.91)  1.00 (0.89;1.12)  1.01 (0.91;1.12)  1.02 (0.91;1.13) | 0.75 (0.47;1.21) **0.50 •• (0.27;0.92) 0.56 • (0.30;1.05)**  0.80 (0.43;1.49)  0.57 (0.26;1.28) 0.67 (0.30;1.51)  0.87 (0.37;2.03)  0.58 (0.20;1.74)  0.59 (0.20;1.74)  **0.35 •• (0.13;0.94)**  0.43 (0.12;1.55)  0.45 (0.12;1.65)  0.93 (0.84;1.02)  0.95 (0.85;1.06)  0.95 (0.85;1.06) |
|  |  |  |  |  |  |  |  |
| Central retinal arteriolar equivalent (µm) n= 2399  Central retinal venular equivalent (µm) n=2399  Retinal arteriolar dilatation (%) n=1881  Retinal venular dilatation (%) n=1911 | Model 1  Model 2  Model 3  Model 1  Model 2  Model 3  Model 1  Model 2  Model 3  Model 1  Model 2  Model 3 | 0.73  (-2.51;3.96)  0.80  (-2.56;4.16)  0.77  (-2.60;4.13)  4.07  (-1.04;9.17)  2.70  (-2.61;8.01)  2.66  (-2.66;7.98)  -0.65  (-1.15;-0.14)  -0.55  (-1.07;-0.02)  -0.57  (-1.10;-0.04)  0.09  (-0.31;0.50)  0.19  (-0.23;0.61)  0.17  (-0.25;0.59) | 0.99  (-2.26;4.24)  1.50  (-1.80;4.81)  1.60  (-1.73;4.92)  4.98  (-0.15;10.10)  4.10  (-1.13;9.34)  4.51  (-0.75;9.78)  -0.07  (-0.57;0.44)  0.03  (-0.50;0.55)  0.00 (-0.52;0.52)  0.41 (0.00;0.82)  0.49 (0.07;0.91)  0.48 (0.06;0.90) | 0.45  (-2.57;3.48)  0.29  (-3.50;4.07)  0.16  (-3.64;3.97)  2.77  (-1.99;7.54)  0.90  (-5.09;6.89)  0.76  (-5.26;6.78)  -0.83  (-1.30;-0.36)  -0.52  (-1.11;0.08)  -0.52  (-1.11;0.08)  -0.01  (-0.39;0.37)  0.41  (-0.06;0.89)  0.40  (-0.08;0.88) | 2.96 (0.45;5.46)  4.29 (1.22;7.36)  4.19 (1.11;7.28)  4.21 (0.26;8.16)  3.57  (-1.28;8.43)  3.57  (-1.31;8.45)  -0.97  (-1.37;-0.58)  -0.74  (-1.22;-0.25)  -0.76  (-1.25;-0.27)  -0.15  (-0.46;0.17)  0.05  (-0.34;0.44)  0.05  (-0.34;0.44) | -0.27  (-4.85;4.32)  -0.70  (-5.42;4.01)  -0.83  (-5.56;3.90)  -0.91  (-8.14;6.32)  -1.41  (-8.86;6.05)  -1.85  (-9.34;5.63)  -0.58  (-1.29;0.13) -0.57  (-1.31;0.17)  -0.57  (-1.31;0.17)  -0.32  (-0.89;0.26)  -0.30  (-0.89;0.29)  -0.31  (-0.91;0.28) | -2.50  (-6.43;1.42)  -4.00  (-8.87;0.87)  -4.03  (-8.93;0.86)  -1.43  (-7.62;4.76)  -2.67  (-10.38;5.03)  -2.81  (-10.56;4.94)  0.14  (-0.47;0.76)  0.22  (-0.55;0.99)  0.24  (-0.53;1.01)  0.14  (-0.36;0.64)  0.36  (-0.25;0.98)  0.35  (-0.27;0.97) |

Sex-specific differences are expressed as linear or logistic regression coefficients (95%-CI) of the dependent variables, which indicate mean differences (βs), odds ratios (ORs) or geometric mean ratios (GMRs) in microvascular complications and retinal measures according to glucose metabolism status. The reference category for prediabetes and type 2 diabetes is normal glucose metabolism status. *ORs † GMRs

Differences between sexes are expressed as linear or logistic regression coefficients (95%-CI) of the interaction terms sex*prediabetes and sex*type 2 diabetes, which indicate differences between women and men in mean differences (βs), women to men ratio of odds ratios (WM-ORs), or women to men ratio of geometric mean ratios (WM-GMRs) in microvascular complications and retinal measures according to glucose metabolism status. *WM-ORs †WM-GMRs Statistically significant differences between the sexes are typed in bold. ••P value <0.05 • P value <0.10.

**Additional file 1: Table S2: Differences within and between sexes in mean differences in microvascular complications and retinal measures according to HbA1c, fasting glucose and 2-h postload glucose (all models)**

|  |  | HbA1c (%)  β, OR or GMR (95%-CI) | | Fasting glucose (mmol/l)  β, OR or GMR (95%-CI) | | 2-h postload glucose (mmol/l)  β, OR or GMR (95%-CI) | | Sex difference  β, WM-OR or WM-GMR (95% CI) | | |
| --- | --- | --- | --- | --- | --- | --- | --- | --- | --- | --- |
|  |  | Women | Men | Women | Men | Women | Men | HbA1c | Fasting glucose | 2-h postload glucose |
| Nephropathy^*^ n=2739  (Yes vs. no)    Albuminuria (mg/24h)†  n=2568  Estimated glomerular  filtration rate (ml/min/1.73m^2^) n=2715 | Model 1  Model 2  Model 3  Model 1  Model 2  Model 3  Model 1  Model 2  Model 3 | 1.82 (1.42;2.33)  1.37 (1.05;1.79)  1.37 (1.05;1.80)  1.21 (1.12;1.30) 1.12 (1.03;1.22)  1.13 (1.04;1.22)  -1.28  (-2.27;-0.28)  -0.10  (-1.22;1.02)  -0.09 (-1.21;1.03) | 1.79 (1.56;2.05)  1.39 (1.18;1.64)  1.38 (1.17;1.62)  1.35 (1.28;1.42) 1.20 (1.14;1.27) 1.20 (1.13;1.27)  -1.01  (-1.70;-0.33)  0.14  (-0.65;0.92)  0.22  (-0.57;1.01) | 1.36 (1.19;1.55)  1.12 (0.96;1.32) 1.12 (0.96;1.32)  1.11 (1.06;1.15) 1.06 (1.01;1.11)  1.06 (1.01;1.11)  -0.26  (-0.82;0.31) 0.66 (0.01;1.31)  0.71 (0.06;1.36) | 1.32 (1.23;1.42)  1.15 (1.06;1.25)   1.15 (1.06;1.25)  1.17 (1.14;1.20) 1.10 (1.07;1.13) 1.10 (1.07;1.13)  -0.28  (-0.64;0.09) 0.39  (-0.02;0.80)  0.41 (0.00;0.82) | 1.11 (1.06;1.17)  1.04 (0.98;1.11)  1.04 (0.98;1.11)  1.02 (1.01;1.04) 1.01 (0.99;1.02)  1.01 (0.99;1.02)  -0.09  (-0.27;0.10) 0.17  (-0.04;0.39)  0.18  (-0.04;0.40) | 1.08 (1.05;1.12)  1.03 (0.99;1.08) 1.03 (0.99;1.08)  1.04 (1.03;1.05) 1.02 (1.01;1.03)  1.02 (1.01;1.03)  -0.12  (-0.28;0.03) 0.13  (-0.04;0.31)  0.14  (-0.04;0.32) | 1.02 (0.77;1.35)  0.99 (0.72;1.35)  1.00 (0.73;1.37)  **0.90 •• (0.82;0.98)**  0.93 (0.84;1.03)  0.94 (0.85;1.04)  -0.26  (-1.47;0.95)  -0.24  (-1.60;1.13)  -0.31  (-1.68;1.06) | 1.03 (0.89;1.19)  0.97 (0.82;1.16)   0.98 (0.82;1.17)  **0.94 •• (0.90;0.99)** 0.96 (0.91;1.02) 0.97 (0.91;1.02)  0.02  (-0.65;0.69) 0.27  (-0.50;1.03)  0.30  (-0.47;1.07) | 1.03 (0.97;1.09)  1.01 (0.94;1.09) 1.01 (0.94;1.09)  **0.98 •• (0.96;1.00)** 0.99 (0.97;1.01) 0.99 (0.97;1.01)  0.03  (-0.21;0.28) 0.04  (-0.24;0.32)  0.04  (-0.24;0.32) |
|  |  |  |  |  |  |  |  |  |  |  |
| Sensory neuropathy^*^ n=2729 (Yes vs. no)    Neuropathic pain^*^ n=2692  (Yes vs. no)  Impaired vibration perception^*^ n=2384 Unilateral vs. no impaired vibration perception  Bilateral vs. no impaired vibration perception  Mean neurothesiometer outcome  on the right and left first toe (Volt) † n=2392 | Model 1  Model 2  Model 3  Model 1  Model 2  Model 3  Model 1  Model 2  Model 3  Model 1  Model 2  Model 3  Model 1  Model 2  Model 3 | 1.76 (1.45;2.15) 1.31 (1.04;1.63)  1.36 (1.09;1.69)  1.86 (1.49;2.31)  1.32 (1.03;1.69) 1.36 (1.07;1.74)  1.31 (0.93;1.87)  1.04 (0.64;1.69)  1.04 (0.65;1.69)  1.75 (1.29;2.37)  1.84 (1.27;2.65)  1.84 (1.28;2.63)  1.08 (1.04;1.13)  1.07 (1.03;1.12)  1.07 (1.03;1.12) | 1.66 (1.46;1.89) 1.41 (1.21;1.64)  1.39 (1.20;1.62)  1.67 (1.44;1.93)  1.39 (1.16;1.66) 1.36 (1.14;1.63)  1.30 (1.06;1.59)  1.16 (0.91;1.48)  1.13 (0.89;1.44)  1.68 (1.39;2.03)  1.42 (1.13;1.79)  1.41 (1.11;1.78)  1.10 (1.07;1.13)  1.08 (1.05;1.12)  1.08 (1.05;1.11) | 1.28 (1.15;1.42) 1.05 (0.93;1.19)  1.07 (0.95;1.21)  1.32 (1.18;1.49)  1.08 (0.94;1.23)  1.10 (0.96;1.26)  1.13 (0.93;1.38)  0.95 (0.71;1.26)  0.95 (0.71;1.27)  1.29 (1.08;1.55)  1.32 (1.06;1.65)  1.33 (1.07;1.66)  1.03 (1.01;1.06)  1.02 (0.99;1.04)  1.02 (0.99;1.04) | 1.24 (1.16;1.32)   1.12 (1.03;1.21)  1.11 (1.03;1.20)  1.24 (1.15;1.33)  1.11 (1.01;1.22)  1.11 (1.01;1.22)  1.14 (1.03;1.26)  1.06 (0.94;1.20)  1.05 (0.93;1.19)  1.25 (1.14;1.38)  1.12 (1.00;1.26)  1.13 (1.00;1.27)  1.05 (1.03;1.06)  1.03 (1.02;1.05)  1.03 (1.02;1.05) | 1.06 (1.02;1.10) 1.01 (0.96;1.06) 1.01 (0.97;1.06)  1.05 (1.01;1.11)  0.99 (0.93;1.05)  1.00 (0.94;1.06)  1.08 (1.01;1.15)  1.05 (0.96;1.14)  1.04 (0.96;1.12)  1.02 (0.93;1.11)  1.05 (0.94;1.17)  1.04 (0.93;1.15)  1.00 (1.00;1.01)  1.00 (1.00;1.01)  1.00 (1.00;1.01) | 1.10 (1.06;1.13) 1.08 (1.04;1.13)  1.08 (1.04;1.12)  1.11 (1.07;1.16)  1.09 (1.04;1.15)  1.09 (1.04;1.15)  1.05 (1.00;1.11)  1.04 (0.98;1.10)  1.03 (0.98;1.09)  1.10 (1.04;1.16)  1.10 (1.03;1.17)  1.09 (1.02;1.16)  1.01 (1.00;1.02)  1.01 (1.00;1.02)  1.01 (1.00;1.02) | 1.06 (0.84;1.35) 0.93 (0.71;1.22)  0.97 (0.74;1.27)  1.11 (0.85;1.45)  0.95 (0.70;1.29)  1.00 (0.74;1.35)  1.01 (0.67;1.52)  0.89 (0.52;1.54)  0.92 (0.54;1.58)  1.04 (0.73;1.49)  1.29 (0.84;1.99)  1.30 (0.85;2.00)  0.99 (0.94;1.03)  0.99 (0.94;1.04)  0.99 (0.94;1.04) | 1.03 (0.91;1.17) 0.94 (0.81;1.09)  0.96 (0.83;1.11)  1.07 (0.93;1.23)  0.97 (0.82;1.14)  0.99 (0.84;1.16)  0.99 (0.79;1.24)  0.89 (0.65;1.22)  0.90 (0.66;1.24)  1.03 (0.84;1.26)  1.18 (0.92;1.52)  1.18 (0.92;1.52)    0.99 (0.96;1.01)  0.98 (0.96;1.01)  0.98 (0.96;1.01) | 0.97 (0.92;1.02) **0.93 •• (0.88;0.99)**  **0.94 •• (0.88;1.00)**  **0.95 • (0.89;1.01)**  **0.90 •• (0.84;0.98)** **0.92 •• (0.85;0.99)**  1.02 (0.94;1.10)  1.01 (0.91;1.11)  1.00 (0.91;1.10)  0.92 (0.83;1.03)  0.95 (0.84;1.08)  0.95 (0.84;1.08)  1.00 (0.99;1.01)  1.00 (0.99;1.01)  1.00 (0.99;1.01) |
|  |  |  |  |  |  |  |  |  |  |  |
| Central retinal arteriolar equivalent (µm) n= 2394  Central retinal venular equivalent (µm) n=2394  Retinal arteriolar dilatation (%) n=1878  Retinal venular dilatation (%) n=1908 | Model 1  Model 2  Model 3  Model 1  Model 2  Model 3  Model 1  Model 2  Model 3  Model 1  Model 2  Model 3 | 0.12  (-1.46;1.71)  -0.15  (-1.94;1.64)  -0.15  (-1.95;1.64)  2.39  (-0.10;4.89)  1.41  (-1.42;4.24) 1.41  (-1.42;4.25)  -0.48  (-0.72;-0.24)  -0.35  (-0.63;-0.07)  -0.34  (-0.62;-0.06)  -0.13  (-0.33;0.07)  -0.02  (-0.25;0.21)  -0.02  (-0.25;0.21) | 1.28 (0.16;2.40)  1.51 (0.20;2.82) 1.44  (0.12;2.75)  1.83 (0.07;3.60)  1.24  (-0.83;3.31) 1.05  (-1.03;3.14)  -0.49  (-0.66;-0.31)  -0.41  (-0.62;-0.20)  -0.41  (-0.62;-0.20)  -0.15  (-0.29;0.00)  -0.11  (-0.29;0.06)  -0.11  (-0.29;0.06) | -0.05  (-0.94;0.84)  -0.28  (-1.32;0.75)  -0.27  (-1.31;0.78)  1.15  (-0.26;2.56)  0.39  (-1.24;2.03) 0.41  (-1.24;2.05)  -0.31  (-0.44;-0.17)  -0.25  (-0.40;-0.09)  -0.24  (-0.40;-0.09)  -0.06  (-0.17;0.05)  0.00  (-0.13;0.12)  0.00  (-0.13;0.13) | 0.27  (-0.32;0.86)  0.31  (-0.36;0.99) 0.29  (-0.39;0.96)  0.23  (-0.70;1.16)  -0.23  (-1.30;0.84)  -0.27  (-1.34;0.81)  -0.22  (-0.32;-0.13)  -0.17  (-0.28;-0.07)  -0.17  (-0.28;-0.07)  -0.07  (-0.15;0.00)  -0.06  (-0.14;0.03)  -0.06  (-0.14;0.03) | -0.15  (-0.46;0.16)  -0.23  (-0.59;0.13)  -0.24  (-0.61;0.12)  -0.05  (-0.54;0.44)  -0.28  (-0.85;0.29)  -0.29  (-0.87;0.29)  -0.04  (-0.09;0.01)  0.00  (-0.06;0.06)  0.00  (-0.06;0.06)  0.01  (-0.03;0.05)  0.03  (-0.02;0.08) 0.03  (-0.02;0.07) | 0.19  (-0.07;0.45)  0.32 (0.02;0.63) 0.33 (0.02;0.63)  0.17  (-0.25;0.58)  0.12  (-0.36;0.60) 0.13  (-0.35;0.61)  -0.07  (-0.11;-0.03)  -0.05  (-0.10;0.00)  -0.06  (-0.10;-0.01)  -0.01  (-0.04;0.02)  0.00  (-0.04;0.04) 0.00  (-0.04;0.04) | -1.16  (-3.09;0.78)  -1.66  (-3.88;0.56)  -1.59  (-3.82;0.63)  0.56  (-2.49;3.61)  0.17  (-3.34;3.68)  0.36 (-3.16;3.88)  0.01  (-0.29;0.31)  0.07  (-0.28;0.42)  0.07  (-0.28;0.42)  0.01  (-0.23;0.26)  0.09  (-0.19;0.38) 0.09  (-0.19;0.38) | -0.32  (-1.39;0.75)  -0.59  (-1.83;0.64)  -0.55  (-1.80;0.69)  0.92  (-0.76;2.61)  0.63  (-1.33;2.58) 0.67  (-1.29;2.64)  -0.08  (-0.24;0.08)  -0.08  (-0.26;0.11)  -0.07  (-0.26;0.12)  0.01  (-0.12;0.14)  0.05  (-0.10;0.20) 0.05  (-0.10;0.21) | -0.34  (-0.74;0.07)  **-0.55 •• (-1.02;-0.08)**  **-0.57 ••**  **(-1.04;-0.09)**  -0.22  (-0.86;0.42)  -0.40  (-1.14;0.35)  -0.42 (-1.17;0.33)  0.03  (-0.03;0.09)  0.05  (-0.02;0.13) 0.06  (-0.02;0.13)  0.02  (-0.04;0.07)  0.03  (-0.03;0.09) 0.03  (-0.03;0.09) |

Sex-specific differences are expressed as linear or logistic regression coefficients (95%-CI) of the dependent variables, which indicate mean differences (βs), odds ratios (ORs) or geometric mean ratios (GMRs) in microvascular complications and retinal measures per one percent point increase in HbA1c or per one mmol/l increase in fasting glucose or 2-h postload glucose. *ORs † GMRs

Differences between sexes are expressed as linear or logistic regression coefficients (95%-CI) of the interaction terms sex*HbA1c, sex*fasting glucose and sex*2-h postload glucose, which indicate differences between women and men in mean differences (βs), women to men ratio of odds ratios (WM-ORs), or women to men ratio of geometric mean ratios (WM-GMRs) in microvascular complications and retinal measures per one percent point increase in HbA1c or per one mmol/l increase in fasting glucose or 2-h postload glucose. *WM-ORs †WM-GMRs Statistically significant differences between the sexes are typed in bold. ••P value <0.05 • P value <0.10.

**Additional file 1: Table S3: Differences between sexes in mean differences in microvascular complications and retinal measures according to glucose metabolism status – with BMI used instead of waist circumference**

|  | Sex difference  β, WM-OR or WM-GMR (95% CI)   Model 2 – waist | | Sex difference,  β, WM-OR or WM-GMR (95% CI)   Model 2 – BMI | |
| --- | --- | --- | --- | --- |
|  | Prediabetes | Type 2 diabetes | Prediabetes | Type 2 diabetes |
| Nephropathy^*^ n=2746  (Yes vs. no)    Albuminuria (mg/24h)†  n=2573  Estimated glomerular  filtration rate (ml/min/1.73m^2^)  n=2722 | 0.66 (0.26;1.68)  0.98 (0.80;1.20)  1.17  (-1.66;4.00) | 1.08 (0.49;2.36)  0.89 (0.72;1.11)  0.47  (-2.45;3.39) | 0.65 (0.25;1.65)  0.98 (0.79;1.20)  1.28  (-1.54;4.11) | 1.03 (0.48;2.23)  0.88 (0.71;1.09)  0.75  (-2.15;3.65) |
|  |  |  |  |  |
| Sensory neuropathy^*^  n=2736 (Yes vs. no)  Neuropathic pain^*^ n=2699 (Yes vs. no)  Impaired vibration perception^*^  n=2390  Unilateral vs. no impaired vibration perception  Bilateral vs. no impaired vibration perception  Mean neurothesiometer outcome  on the right and left first toe (Volt)†  n=2398 | 1.13 (0.59;2.17)  1.40 (0.56;3.53)  0.96 (0.32;2.90)  0.67 (0.15;3.04)  1.01 (0.91;1.12) | **0.50 •• (0.27;0.92)**  0.57 (0.26;1.28)  0.58 (0.20;1.74)  0.43 (0.12;1.55)  0.95 (0.85;1.06) | 1.11  (0.58;2.11)  1.43 (0.57;3.57)  0.92 (0.30;2.76)  0.61 (0.14;2.78)  1.01 (0.90;1.12) | **0.50 •• (0.27;0.91)**  0.62 (0.28;1.35)  0.56 (0.19;1.63)  0.37 (0.10;1.32)  0.95 (0.85;1.06) |
|  |  |  |  |  |
| Central retinal arteriolar equivalent (µm) n= 2399  Central retinal venular equivalent (µm) n=2399  Retinal arteriolar dilatation (%) n=1881  Retinal venular dilatation  (%) n=1911 | -0.70  (-5.42;4.01)  -1.41  (-8.86;6.05)  -0.57  (-1.31;0.17)  -0.30  (-0.89;0.29) | -4.00  (-8.87;0.87)  -2.67  (-10.38;5.03)  0.22  (-0.55;0.99)  0.36  (-0.25;0.98) | -0.83  (-5.53;3.88)  -1.26  (-8.70;6.18)  -0.59  (-1.33;0.15)  -0.28  (-0.87;0.31) | -4.42  (-9.24;0.40)  -2.52  (-10.14;5.11)  0.20  (-0.57;0.96)  0.41  (-0.20;1.02) |

Differences between sexes (main model: model 2) are expressed as linear or logistic regression coefficients (95%-CI) of the interaction terms sex*prediabetes and sex*type 2 diabetes, which indicate differences between women and men in mean differences (βs), women to men ratio of odds ratios (WM-ORs) or women to men ratio of geometric mean ratios (WM-GMRs) in microvascular complications and retinal measures according to glucose metabolism status. *WM-ORs †WM-GMRs Statistically significant differences between the sexes are typed in bold. ••P value <0.05 • P value <0.10.

**Additional file 1: Table S4: Differences within and between sexes in mean differences in microvascular complications and retinal measures according to glucose metabolism status – premenopausal women excluded**

|  |  | Prediabetes  β, OR or GMR (95%-CI) Excl. Premenopausal women N=3017 | | Type 2 diabetes  β, OR or GMR (95%-CI)  Excl. Premenopausal women N=3017 | | Sex difference  β, WM-OR or WM-GMR (95% CI)   Excl. Premenopausal women N=3017 | |
| --- | --- | --- | --- | --- | --- | --- | --- |
|  |  | Women | Men | Women | Men | Prediabetes | Type 2 diabetes |
| Nephropathy ^*^  n=2427 (Yes vs. no)    Albuminuria (mg/24h)† n=2284  Estimated glomerular  filtration rate (ml/min/1.73m^2^) n=2411 | Model 1  Model 2  Model 3  Model 1  Model 2  Model 3  Model 1  Model 2  Model 3 | 1.13 (0.55;2.32)  0.72 (0.33;1.57) 0.73 (0.33;1.59)  1.05 (0.89;1.24) 0.96 (0.81;1.14) 0.96 (0.81;1.14)  -0.10  (-2.36;2.15) 1.55  (-0.74;3.83) 1.54  (-0.74;3.82) | 1.46 (0.87;2.43)  0.96 (0.56;1.65)  0.99 (0.58;1.71)  1.11 (0.96;1.29) 0.94 (0.82;1.09)  0.96 (0.83;1.11)  -0.85  (-2.82;1.12) 0.95  (-1.02;2.92) 0.87  (-1.10;2.84) | 3.43 (2.05;5.75)  1.51 (0.77;2.98) 1.54 (0.77;3.06)  1.32 (1.13;1.54) 1.14 (0.94;1.37) 1.15 (0.96;1.39)  -3.07  (-5.12;-1.02) 0.22  (-2.27;2.72) 0.42  (-2.08;2.93) | 3.52 (2.44;5.08)  1.58 (1.01;2.46)  1.58 (1.00;2.47)  1.82 (1.62;2.04) 1.32 (1.16;1.52)  1.34 (1.17;1.53)  -1.93  (-3.47;-0.40) 1.00  (-0.82;2.82) 1.12  (-0.71;2.94) | 0.78 (0.32;1.88)  0.75 (0.29;1.92) 0.73 (0.28;1.90)  0.94 (0.75;1.17) 1.02 (0.81;1.27)  1.00 (0.80;1.26)  0.75  (-2.24;3.74) 0.60  (-2.42;3.61) 0.67  (-2.34;3.69) | 0.98 (0.52;1.84)  0.96 (0.42;2.15) 0.98 (0.43;2.22)  **0.73 •• (0.60;0.88)** 0.86 (0.68;1.08) 0.86 (0.69;1.09)  -1.14  (-3.69;1.42)  -0.78 (-3.87;2.31)  -0.69  (-3.79;2.41) |
|  |  |  |  |  |  |  |  |
| Sensory neuropathy^*^ n=2417  (Yes vs. no)    Neuropathic pain^*^ n=2388 (Yes vs. no)  Impaired vibration perception^*^ n=2108  Unilateral vs. no impaired vibration perception  Bilateral vs. no impaired vibration perception  Mean neurothesiometer outcome on the right and left first toe (Volt)† n=2116 | Model 1  Model 2  Model 3  Model 1  Model 2  Model 3  Model 1  Model 2  Model 3  Model 1  Model 2  Model 3  Model 1  Model 2  Model 3 | 1.38 (0.87;2.18) 1.04 (0.64;1.71) 1.08 (0.65;1.77)  1.44 (0.80;2.62)  1.03 (0.54;1.96) 1.06 (0.55;2.03)  1.46 (0.62;3.45)  1.09 (0.43;2.74)  1.09 (0.43;2.75)  0.76 (0.25;2.34)  0.68 (0.21;2.20)  0.73 (0.22;2.39)  1.02 (0.93;1.12)  1.01 (0.93;1.10)  1.01 (0.93;1.11) | 1.13 (0.72;1.78) 0.96 (0.60;1.54) 0.91 (0.56;1.46)  1.05 (0.52;2.14)  0.85 (0.41;1.77) 0.84 (0.41;1.76)  1.24 (0.63;2.44)  1.06 (0.53;2.14)  1.03 (0.51;2.08)  1.22 (0.48;3.07)  0.99 (0.38;2.55)  0.84 (0.32;2.20)  1.04 (0.96;1.13)  1.03 (0.95;1.11)  1.03 (0.95;1.11) | 2.43 (1.66;3.55) 1.23 (0.74;2.06) 1.34  (0.80;2.24)  2.87 (1.79;4.59)  1.36 (0.71;2.60)  1.53 (0.80;2.93)  1.92 (0.93;3.95)  1.05 (0.40;2.78)  1.01 (0.39;2.64)  1.57 (0.71;3.46)  1.38 (0.48;3.97)  1.36 (0.47;3.97)  1.12 (1.03;1.21)  1.07 (0.97;1.17)  1.06 (0.97;1.16) | 3.34 (2.44;4.58) 2.46 (1.67;3.63) 2.37 (1.60;3.50)  3.92 (2.52;6.11)  2.51 (1.47;4.28) 2.42 (1.41;4.15)  2.09 (1.27;3.43)  1.69 (0.93;3.08)  1.61 (0.89;2.93)  4.31 (2.35;7.91)  3.10 (1.51;6.35)  2.98 (1.43;6.21)  1.19 (1.12;1.27)  1.13 (1.06;1.22)  1.13 (1.05;1.21) | 1.22 (0.64;2.32) 1.08 (0.55;2.15) 1.19  (0.60;2.37)  1.37 (0.54;3.47)  1.21 (0.46;3.20)  1.26 (0.47;3.35)  1.17 (0.39;3.50)  1.02 (0.32;3.25)  1.06 (0.33;3.38)  0.63 (0.15;2.67)  0.69 (0.15;3.11)  0.87 (0.19;4.01)  0.98 (0.87;1.11)  0.98 (0.87;1.10)  0.99 (0.88;1.11) | 0.73 (0.44;1.19) **0.50 •• (0.26;0.95) 0.57 • (0.30;1.08)**  0.73 (0.38;1.39)  0.54 (0.23;1.26)  0.63 (0.27;1.47)  0.92 (0.38;2.21)  0.62 (0.20;1.95)  0.63 (0.20;1.95)  **0.36 •• (0.13;0.98)**  0.44 (0.12;1.60)  0.46 (0.12;1.67)  0.94 (0.84;1.04)  0.94 (0.84;1.06)  0.94 (0.83;1.05) |
|  |  |  |  |  |  |  |  |
| Central retinal arteriolar equivalent (µm) n= 2124  Central retinal venular equivalent (µm) n=2124  Retinal arteriolar dilatation (%) n=1662  Retinal venular dilatation (%) n=1687 | Model 1  Model 2  Model 3  Model 1  Model 2  Model 3  Model 1  Model 2  Model 3  Model 1  Model 2  Model 3 | 0.64  (-3.01;4.30)  0.50  (-3.29;4.29)  0.47  (-3.33;4.26)  5.08  (-0.69;10.86)  3.12  (-2.87;9.11)  3.07  (-2.93;9.08)  -0.51  (-1.08;0.07) -0.36  (-0.96;0.23) -0.39  (-0.99;0.21)  0.13  (-0.34;0.60) 0.26  (-0.22;0.75) 0.22  (-0.26;0.71) | 0.99  (-2.25;4.24)  1.50  (-1.80;4.81)  1.60  (-1.73;4.92)  4.98  (-0.14;10.10)  4.10  (-1.12;9.33) 4.51  (-0.75;9.77)  -0.07  (-0.58;0.44) 0.03  (-0.50;0.55) 0.00  (-0.53;0.53)  0.41  (-0.01;0.83) 0.49 (0.06;0.92)  0.48 (0.05;0.91) | 0.37  (-2.93;3.67)  -0.56  (-4.69;3.57) -0.61  (-4.77;3.55)  2.65  (-2.55;7.86)  -0.91  (-7.44;5.62)  -0.94  (-7.52;5.64)  -0.88  (-1.40;-0.37) -0.51  (-1.15;0.14) -0.52  (-1.17;0.13)  0.06  (-0.36;0.49)  0.54 (0.01;1.07)  0.55 (0.02;1.08) | 2.96 (0.46;5.46)  4.29 (1.22;7.35)  4.19 (1.11;7.28)  4.21 (0.26;8.15)  3.57  (-1.27;8.42)  3.57  (-1.31;8.45)  -0.97  (-1.37;-0.57) -0.74  (-1.22;-0.25)  -0.76  (-1.25;-0.27)  -0.15  (-0.47;0.18) 0.05  (-0.35;0.45)  0.05  (-0.35;0.45) | -0.35  (-5.24;4.54)  -1.00  (-6.03;4.02)  -1.13  (-6.17;3.92)  0.11  (-7.61;7.82)  -0.98  (-8.94;6.97)  -1.44  (-9.42;6.54)  -0.44  (-1.21;0.33) -0.39  (-1.18;0.41)  -0.39  (-1.19;0.41)  -0.28  (-0.91;0.35)  -0.23  (-0.88;0.42)  -0.26  (-0.91;0.39) | -2.59  (-6.73;1.55)  -4.85  (-9.99;0.29)  **-4.80 • (-9.98;0.38)**  -1.56  (-8.09;4.98)  -4.48  (-12.62;3.65) -4.51  (-12.70;3.68)  0.09  (-0.57;0.74) 0.23  (-0.58;1.04) 0.24  (-0.58;1.05)  0.21  (-0.32;0.75) 0.49  (-0.17;1.15)  0.50  (-0.16;1.16) |

Sex-specific differences are expressed as linear or logistic regression coefficients (95%-CI) of the dependent variables, which indicate mean differences (βs), odds ratios (ORs) or geometric mean ratios (GMRs) in microvascular complications and retinal measures according to glucose metabolism status. The reference category for prediabetes and type 2 diabetes is normal glucose metabolism status. *ORs † GMRs

Differences between sexes are expressed as linear or logistic regression coefficients (95%-CI) of the interaction terms sex*prediabetes and sex*type 2 diabetes, which indicate differences between women and men in mean differences (βs), women to men ratio of odds ratios (WM-ORs) or women to men ratio of geometric mean ratios (WM-GMRs) in microvascular complications and retinal measures according to glucose metabolism status. *WM-ORs †WM-GMRs Statistically significant differences between the sexes are typed in bold. ••P value <0.05 • P value <0.10.

**Additional file 1: Table S5: Differences within and between sexes in mean differences in neuropathy according to glucose metabolism status – with additional adjustment for metformin use (model 2 and 3)**

|  |  | Prediabetes  OR or GMR  (95%-CI) | | Type 2 diabetes  OR or GMR  (95%-CI) | | Sex difference,  WM-OR or WM-GMR (95% CI) | |
| --- | --- | --- | --- | --- | --- | --- | --- |
|  |  | Women | Men | Women | Men | Prediabetes | Type 2 diabetes |
| Sensory neuropathy^*^  n=2736 (Yes vs. no)    Neuropathic pain^*^ n=2699 (Yes vs. no)    Impaired vibration perception^*^ n=2390  Unilateral vs. no impaired vibration perception  Bilateral vs. no impaired vibration perception    Mean neurothesiometer outcome on the right and left first toe (Volt)^†^  n=2398 | Model 1  Model 2  Model 3  Model 1  Model 2  Model 3  Model 1  Model 2  Model 3  Model 1  Model 2  Model 3  Model 1  Model 2  Model 3 | 1.54 (1.02;2.33) 1.09 (0.69;1.71)  1.12 (0.71;1.76)  1.75 (1.04;2.94)  1.20 (0.68;2.11) 1.24 (0.70;2.20)  1.42 (0.64;3.16)  1.02 (0.43;2.41) 1.01 (0.43;2.38)  0.74 (0.24;2.27)  0.67 (0.21;2.16) 0.72 (0.22;2.33)  1.03 (0.95;1.12)  1.04 (0.96;1.12)  1.04 (0.97;1.12) | 1.13 (0.72;1.78)  0.96 (0.60;1.54)  0.90 (0.56;1.45)  1.05 (0.52;2.14)  0.85 (0.41;1.77) 0.85 (0.41;1.77)  1.24 (0.63;2.44)  1.06 (0.53;2.14) 1.03 (0.51;2.07)  1.22 (0.48;3.07)  0.99 (0.38;2.55) 0.84 (0.32;2.20)  1.04 (0.96;1.13)  1.03 (0.96;1.11) 1.03 (0.95;1.11) | 2.52 (1.77;3.60) 1.64 (0.90;2.97)  1.78 (0.97;3.24)  3.14 (2.03;4.85)  1.88 (0.92;3.86) 2.16 (1.04;4.51)  1.82 (0.92;3.60)  1.17 (0.40;3.43) 1.13 (0.39;3.29)  1.50 (0.68;3.31)  1.25 (0.33;4.71) 1.34 (0.35;5.18)  1.11 (1.03;1.19)  1.13 (1.01;1.26) 1.13 (1.01;1.27) | 3.34 (2.44;4.58) 2.62 (1.64;4.21) 2.52 (1.56;4.06)  3.92 (2.52;6.11)  2.46 (1.30;4.65) 2.30 (1.20;4.40)  2.09 (1.27;3.43)  1.75 (0.85;3.64) 1.73 (0.83;3.59)  4.31 (2.35;7.91)  2.93 (1.27;6.78) 2.94 (1.25;6.93)  1.19 (1.12;1.27)  1.10 (1.00;1.20) 1.09 (1.00;1.20) | 1.36 (0.74;2.52)  1.13 (0.59;2.17) 1.24 (0.64;2.39)  1.67 (0.69;4.01)  1.41 (0.56;3.54) 1.47 (0.58;3.73)  1.14 (0.40;3.26)  0.96 (0.32;2.90) 0.99 (0.33;2.99)  0.61 (0.14;2.59)  0.67 (0.15;3.04) 0.85 (0.19;3.91)  1.00 (0.89;1.12)  1.01 (0.91;1.12) 1.02 (0.91;1.13) | 0.75 (0.47;1.21) 0.62 (0.29;1.33) 0.71 (0.33;1.52)  0.80 (0.43;1.49)  0.77 (0.29;2.01) 0.94 (0.35;2.51)  0.87 (0.37;2.03)  0.67 (0.18;2.45) 0.65 (0.18;2.38)  **0.35 •• (0.13;0.94)**  0.43 (0.09;2.05) 0.46 (0.09;2.26)  0.93 (0.84;1.02)  1.03 (0.89;1.19) 1.03 (0.90;1.19) |

Sex-specific differences are expressed as linear or logistic regression coefficients (95%-CI) of the dependent variables, which indicate mean differences (βs), odds ratios (ORs) or geometric mean ratios (GMRs) in neuropathy-related variables according to glucose metabolism status. The reference category for prediabetes and type 2 diabetes is normal glucose metabolism status. *ORs † GMRs

Differences between sexes are expressed as linear or logistic regression coefficients (95%-CI) of the interaction terms sex*prediabetes and sex*type 2 diabetes, which indicate differences between women and men in mean differences (βs), women to men ratio of odds ratios (WM-ORs), or women to men ratio of geometric mean ratios (WM-GMRs) in neuropathy-related variables according to glucose metabolism status. *WM-ORs †WM-GMRs Statistically significant differences between the sexes are typed in bold. ••P value <0.05 • P value <0.10.
